# Mass Spectrometry-Based Proteomics Approach Characterizes the Dual Functionality of miR-328 in Monocytes

**DOI:** 10.3389/fphar.2019.00640

**Published:** 2019-06-05

**Authors:** Meike J. Saul, Anett B. Hegewald, Anne C. Emmerich, Elena Ossipova, Marc Vogel, Isabell Baumann, Kim Kultima, Johan Lengqivst, Dieter Steinhilber, Per Johan Jakobsson

**Affiliations:** ^1^Department of Biology, Technische Universität Darmstadt, Darmstadt, Germany; ^2^Institute of Pharmaceutical Chemistry, Goethe Universität Frankfurt, Frankfurt, Germany; ^3^Rheumatology Unit, Department of Medicine, Solna, Karolinska Institutet, Karolinska University Hospital at Solna, Stockholm, Sweden; ^4^Clinical Chemistry Research Group, Department of Medical Sciences, Uppsala University, Uppsala, Sweden

**Keywords:** miR-328, noncanonical miR function, proteomics, TLR2, NOX2, p53, inflammation

## Abstract

MicroRNAs (miRs) are small noncoding RNAs which control the expression of target genes by either translational repression or RNA degradation, known as canonical miR functions. The recent discovery that miR-328 has a noncanonical function and can activate gene expression by antagonizing the activity of heterogeneous ribonuclear protein E2 (hnRNP E2) opens an unexplored and exciting field of gene expression regulation. The global importance of such noncanonical miR function is not yet known. In order to achieve a better understanding of the new miR activity, we performed a compartment specific tandem mass tag (TMT)-based proteomic analysis in differentiated MonoMac6 (MM6) cells, to monitor gene expression variations in response to miR-328 knockdown. We identified a broad spectrum of novel potential miR-328/hnRNP E2 and miR-328 targets involved in regulation of compartment specific cellular processes, such as inflammation or RNA splicing. This study provides first insights of the global significance of noncanonical miR function.

## Introduction

MicroRNAs (miRs) are a family of small noncoding RNAs of about 21–24 nucleotides that regulate a wide spectrum of cellular biological processes including inflammation and cancerogenesis (Croce, 2009; Ochs et al., 2011; Ochs et al., 2014). To date, more than 2,500 miRs have been identified (http://www.mirbase.org) while the function of many miR remains unclear.

MiRs are generated by enzymatic processes from precursor transcripts and assembled with the RNA interference silencing complex (RISC). Then, they are directed to their binding sites in the 3′ untranslated region (UTR) of the target messenger RNA (mRNA) and mediate either translational repression or degradation of their target transcript (Croce, 2009; Ochs et al., 2011; Ochs et al., 2014). It has long been a dogma that miRs loaded in RISCs bind to their target mRNA through specific base pairing thereby reducing gene expression at posttranscriptional level. However, there is recent evidence that miRs are also able to activate gene expression *via* a novel miR function described as noncanonical function. MiRs can bind to RNA binding proteins (RBPs) sequestering them away from their target mRNAs in a RISC independent manner (Eiring et al., 2010). Such a function was described for the first time for miR-328. It acts as an RNA decoy to the heterogeneous nuclear ribonucleoprotein E2 (hnRNP E2), a global posttranscriptional regulator (Eiring et al., 2010; Saul et al., 2016). Recently, we found that apart from its function as translational repressor, hnRNP E2 can bind to C-rich sequences within 5´UTR located introns acting as splicing regulator in monocytes (Saul et al., 2016). During myeloid cell differentiation, miR-328 is significantly induced and antagonizes hnRNP E2 activity which results in the upregulation of hnRNP E2 target genes. One representative gene controlled by the hnRNP E2/miR-328 balance is the calcium binding protein S100A9 which plays an important role in cell differentiation, inflammatory response and oxidative stress response of monocytes. During monocyte maturation, miR-328 increases reactive oxygen species (ROS) production as well as adhesion and migration ability of monocytes by modulating the monocytic surface marker cluster of differentiation molecule 11 (CD11b). Recently, we were able to attribute this effect to the novel noncanonical miR-328 function (Saul et al., 2016). Overall, these discoveries reveal intriguing novel functions of miRs and open up a yet unexplored and exciting field of miR research. In order to achieve a better understanding of the dual activity of miR-328, we performed a quantitative tandem mass tags (TMT)-based proteomic study in differentiated MonoMac6 (MM6) cells to monitor gene expression variations in response to miR-328 knockdown. This study identified a broad spectrum of novel potential miR-328/hnRNP E2 and miR-328 targets involved in important cellular processes such as inflammation, p53 signalling or mRNA splicing. For the first time our results give an impression of the global significance and distribution of the noncanonical miR-328 function which will facilitate new strategies in miR research.

## Materials and Methods

### Cell Culture

MonoMac6 (MM6) cells were obtained from DSMZ (DSMZ no. ACC124) and grown in RPMI-1640 medium supplemented with 10% (v/v) fetal calf serum (FCS, Biochrom AG), 100 μg/ml streptomycin (PAA), 100 U/ml penicillin (PAA), 1× non essential amino acids (Sigma Aldrich), 10 μg/ml insulin, 1 mM oxaloacetate (AppliChem), and 1 mM sodium pyruvate (PAA). Cell culture was carried out in a humidified atmosphere of 5% CO_2_ at 37°C. MM6 cells were differentiated with 1 ng/ml TGFβ (PeproTech) and 50 nM calcitriol (Sigma Aldrich) at 37°C, 6% CO_2_. HeLa cells were obtained from DSMZ (DSMZ no. ACC57). They were grown in Dulbecco’s modified Eagle’s medium (DMEM) supplemented with 10% (v/v) FCS, 100 μg/ml streptomycin, and 100 U/ml penicillin. Cell culture was carried out in a humidified atmosphere of 5% CO_2_ at 37°C.

### miR-328 Knockdown in MM6 Cells

As previously described in Saul et al. (2016), we used 2 pmol/μl of a 3′-cholesterol-tagged ON TARGET siRNA-miR-328 (GGGAGAAAGUGCAUACAGC-3′-Chl) or control siRNA (5′-UCUCUCACAACGGGCAUUU-3′-Chl), which was directly added to MM6 cell culture medium. Both siRNAs were synthesized by GE Dharmacon. The efficiency of miR-328 knockdown was assessed by quantitative polymerase chain reaction (qPCR) in 4 days differentiated MM6 cells and revealed a knockdown efficiency of around 85% for both biological replicates, which were used for proteomics study.

### Fraction Preparation

The soluble and microsomal fractionation was performed as previously described in (Ochs et al., 2013; Saul et al., 2016). The protein content in Western blot samples was determined by Bradford assay (BioRad Laboratories), for proteomics samples the protein amount was determined by Pierce BCA Protein Assay (Thermo Fisher Scientific) following manufacturer’s instructions.

### Trypsin Digestion and TMT Labeling

Twenty-five micrograms of protein were taken from microsomal and soluble fraction, respectively, and subjected to disulfide reduction by addition of 5 μl of DTT of 200 mM DTT (Sigma Aldrich) in 100 mM NH_4_HCO_3_ (Carl Roth) for 30 min at 56°C. The sulfhydryl alkylation was performed by adding 4 μl of 1 M iodoacetamide (Sigma Aldrich) in 100 mM NH_4_HCO_3_ at room temperature for 1 h in a dark room. Trypsin (modified sequencing grade, Promega) was added (1:30, trypsin/protein), and the samples were incubated at 37°C overnight. Tandem Mass Tag^™^ 6-plex (TMTsixplex^™^) Isobaric Label Reagents (ThermoFisher Scientific) were used for peptide labeling according to the instruction by the manufacturer. As internal standards we pooled 10 µg of each soluble and microsomal fraction and 25 µg of total pool were labeled with TMTsixplex^™^ label reagent 130 and 131, respectively. All separately labeled samples were pooled into final two TMT sets, one containing soluble fractions and one containing microsomal fraction. Excess TMTsixplex^™^ reagent was removed from the pooled samples using an SCX-cartridge (StrataSCX, Phenomenex), and the eluates were dried in SpeedVac. Next, we performed peptide prefractionation as described in Cao et al. (2012). Briefly, TMT-labeled protein digests were separated over a 45-min gradient (3–55% B) on a 2.1 × 250 mm XBridge BEH300 C18 column (Waters) at the flow rate of 200 µl/min. A- and B-buffers consisted of 20 mM ammonia in MilliQ-grade water and 20 mM ammonia in 80% acetonitrile (ACN), respectively. Fractions were collected per minute and the fractions covering the peptide elution range were combined into 12 final fraction. Obtained fractions were evaporated in SpeedVac and stored at −20°C until Liquid chromatography tandem mass spectrometry (LC-MS/MS) analysis.

### Mass Spectrometry

Online LC-MS was performed using a hybrid Q-Exactive mass spectrometer (ThermoFisher Scientific). Samples were trapped on an Acclaim^™^ PepMap^™^ 100 C18 desalting column (ThermoFisher Scientific), and separated on a 50-cm-long EASY-spray column (50 cm × 75 μm ID, PepMap RSLC C18, 2-μm particles, 100-Å pore size, ThermoFisher Scientific) installed on to the EASY-Spray Series ion source. Solvent A was 97% water, 3% ACN, 0.1% formic acid; and solvent B was 5% water, 95% acetonitrile, 0.1% formic acid. At a constant flow of 0.25 μl min^−1^, the curved gradient went from 2% B up to 48% B in 55 min, followed by a steep increase to 100% B in 5 min. FTMS master scans with 70,000 resolution (and mass range 400–1,200 m/z) were followed by data-dependent MS/MS (17,500 resolution) on the top 10 ions using higher-energy collision dissociation (HCD) at 31% normalized collision energy. Precursors were isolated with a 2 *m/z* window. Automatic gain control (AGC) targets were 3e6 for MS1 and 2e5 for MS2. Maximum injection times were 250 ms for MS1 and 200 ms for MS2. The entire duty cycle lasted ∼2.5 s. Dynamic exclusion was used with 20-s duration. Precursors with unassigned charge state or charge state 1 were excluded. An underfill ratio of 1% was used.

### Data Analysis

Acquired MS raw files were searched using Sequest-Percolator under the software platform Proteome Discoverer 1.4.1.14 (Thermo Fisher Scientific) against human Uniprot database (release 01.12.2015) and filtered to a 1% FDR cutoff. We used a precursor ion mass tolerance of 10 ppm, and product ion mass tolerances of 0.02 Da for HCD-FTMS and 0.8 Da for CID-ITMS. The algorithm considered tryptic peptides with maximum two missed cleavages; carbamidomethylation (C), TMT 6-plex (K, N-term) as fixed modifications, and oxidation (M) as dynamic modifications. Quantification of reporter ions was done by Proteome Discoverer on HCD-FTMS tandem mass spectra using an integration window tolerance of 10 ppm. Only unique peptides in the data set were used for quantification. Biological context and molecular networks were analyzed by Ingenuity Pathway Analysis (Ingenuity Systems, www.ingenuity.com) and STRING database platform (Szklarczyk et al., 2017).

### Western Blotting

Western blot analysis was performed as previously described (Ochs et al., 2013). The membranes were incubated with primary antibodies that recognize TLR2 (D7G9Z, Cell signaling), NOX2 (611414, BD Bioscience), β-actin (sc1616, Santa Cruz), HMGB1 antibody (2G7, mouse IgG2b, noncommercial antibody), S100A9 (ab63818, Abcam), and secondary near infrared dye-conjugated secondary antibodies (IRDye, Li-COR Bioscience). Visualization and quantitative analysis were carried out with an Odyssey Infrared Imaging System (LICOR Biosciences). Odyssey NEWBLOT Nitro Stripping buffer (LI-COR Biosciences) was used for membrane stripping according to manufacturer’s instructions.

### Plasmid Constructs

The luciferase reporter gene constructs containing either the NOX2- or the TLR2 3’UTR were constructed by standard restriction ligation. The 3’UTR was PCR amplified from cDNA out of MM6 cells by Q5 polymerase (New England Biolabs). For the amplification of the NOX2 3’UTR the oligonucleotides NOX_NotI_fwd (5’-AAAAAGCGGCCGCCTTGTCTCTTCCATGAGGAA-3’) and NOX_HindIII_rev (5’-AAAAAAAGCTTGAAAGCTCATTCATTTTAATAG-3’) were used. The TLR2 3’UTR was amplified by using the oligonucleotides TLR2_fwd (5’-GGCCGCGTTCCCATATTTAAG-3’) and TLR2_rev (5’-AGCTTTTCTCATCCTGTAAAG-3’). The oligonucleotides contained the restriction sites NotI and HindIII to clone the DNA fragments into the vector pDLAAG (kindly provided by J. Weigand, TU Darmstadt) (Kemmerer and Weigand, 2014) downstream of the luc2 gene. To verify miR-328 binding the seed region was mutated in both 3’UTRs. The mutated NOX2 3’UTR was constructed by site-directed mutagenesis and Golden Gate (GG) Assembly. Therefore, two DNA fragments were created by PCR using the primer pair NOX2_GG-fwd (5’-ACTGGAAGACTCAATTTCATTAAGGCCAAGAAGGGC-3’) and NOX2_mut-rev (5’-ACTGGAAGACCTGCTGTATTAGTAAACTGGAGTATGCTC-3’) and the primer pair NOX2_mut-fwd (5’-CTGGAAGACCTCAGCGCTGTAACTGCCTTGGATGTTCTTTCTACAGAAGAATATTGG-3’) and NOX2_GG-rev (5’-ACTGGAAGACTCGATCCACTTTGGGCAGGAAATTAGTCTGC-3’). The two DNA fragments were Golden Gate cloned (Engler et al., 2008) into the vector pJBL2807-empty, a for Golden Gate Assembly modified version of pJBL2807 vector (Chappell et al., 2015). Afterward, the mutated NOX2 3’UTR was PCR amplified (NOX2_mut-fwd and NOX2-rev: 5’-TAAGGGCTAGCTGGAGAAGACCACTTTGGGCAGGAAATTAGTCTGC-3’) and cloned into the vector pDLAAG by standard restriction ligation using the unique restriction sites NotI and NheI. To create a modified TLR2 3’UTR with a mutated miR-328 binding site, an overlap extension PCR with two fragments was performed with the oligonucleotides 3’UTR-fwd (5’-TCATTAAGGCCAAGAAGGGC-3’) and TLR2_mut-rev (5’-GCCAGTTGCTACAGATTACAGTCAATCCCTTATATACATGGGTTCTGCATCCATGAAG-3’) for fragment 1 and the oligonucleotides TLR2_mut-fwd (5’-ATGTATATAAGGGATTGACTGTAATCTGTAGCAACTGGC-3’) and 3UTR-rev (5’-TGTGGTATGGCTGATTATGATCC-3’) for fragment 2. Afterward, the two fragments were used for an overlap extension PCR containing the oligonucleotides 3’UTR-fwd and 3’UTR-rev. The DNA fragment was cloned into the vector pDLAAG by standard restriction ligation using the unique restriction sites NotI and HindIII. All plasmids and sequences are available upon request.

### Transfection of miR-328 Mimic

Twenty-four hours prior to transfection, HeLa cells were seeded at a density of 5×10^5^ per well in a six-well plate; 5 nmol MISSION^®^ miR-328 mimics (HMI0483, Sigma Aldrich) or negative control (HMC0002, Sigma Aldrich) was transfected using Lipofectamine 2000^®^ (Invitrogen) according to the manufacturer’s instructions. The transfection efficiency was determined by qPCR according to Saul et al. (2016).

### Luciferase Reporter Gene Assay

Twenty-four hours prior to transfection, 4×10^4^ HeLa cells per well were seeded in 24-well plates; 400 ng/well of TLR2 or NOX2 constructs and 5 nmol miR-328 mimics and control mimics were used for transfection with Lipofectamine 2000^®^ (Invitrogen) according to the manufacturer’s instructions. After 24 h, cells were assayed for luciferase activity using the Dual-Glo^™^ Stop and Glow Luciferase Assay System (Promega) with a TECAN infinite M200 reader. Renilla luciferase activity was used to normalize the luciferase activity to the transfection efficacy.

### Statistics

Results are given as mean + SEM of minimum three independent experiments. Statistical analysis was carried out by Student’s paired or unpaired t test (two-tailed). Differences were considered as significant for p < 0.05 (indicated as *p < 0.05, **p < 0.01) using GraphPad Prism 5.0.

## Results

### Analysis of Protein Expression Changes in Response to miR-328 Knockdown in MM6 Cells

**Figure 1:**
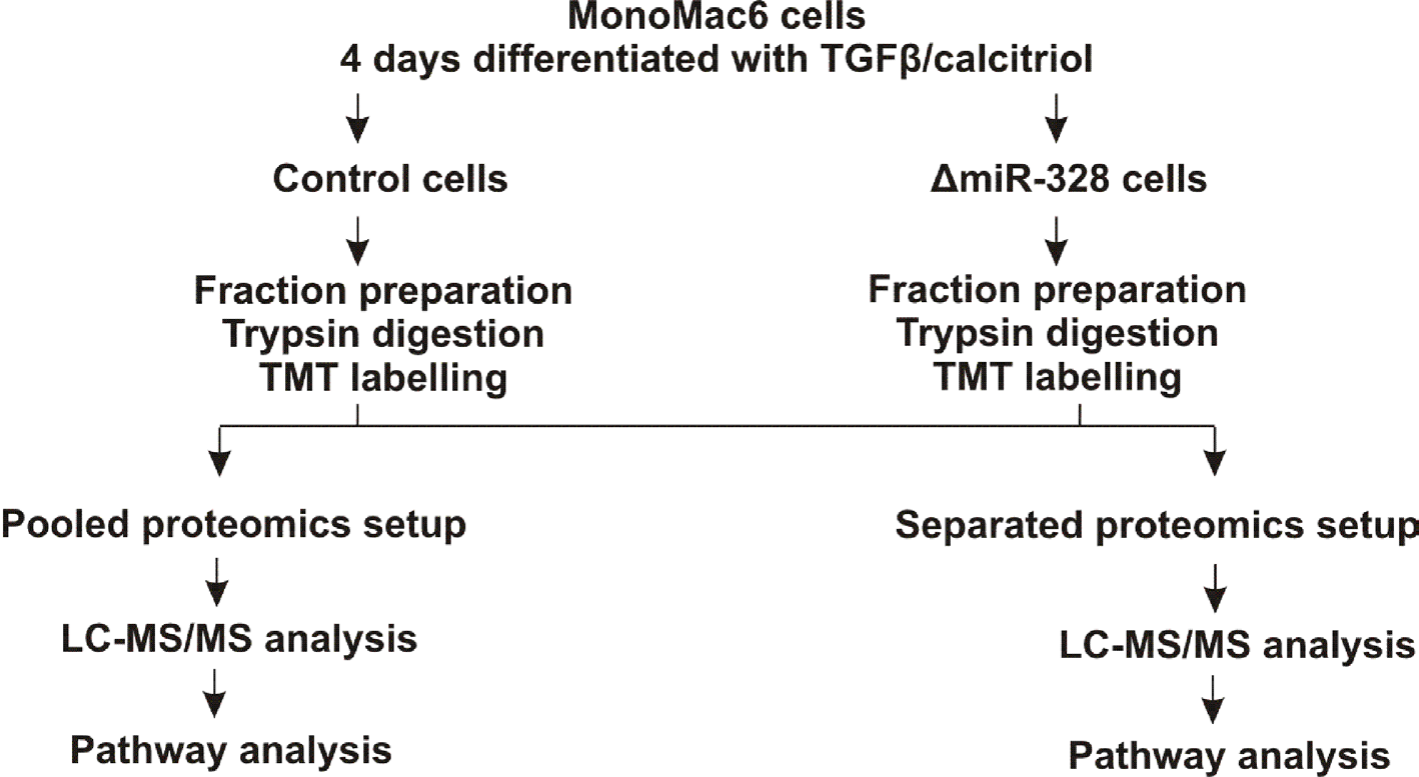
Experimental schema for the tandem mass tags (TMT) based proteomics study. MM6 cells were treated either with 2 pmol/µl control siRNA or miR-328 siRNA and differentiated for 4 days with 1 ng/ml TGFβ and 50 nM calcitriol. Soluble and microsomal fractions were prepared and each fraction was digested with trypsin. The peptides were labelled with TMT reagents. Pooled setup: Samples from microsomal and soluble fraction were mixed together and analyzed simultaneously by LC-MS/MS. Separated setup: Samples from microsomal and soluble fraction were analyzed separately by LC-MS/MS.

**Figure 2:**
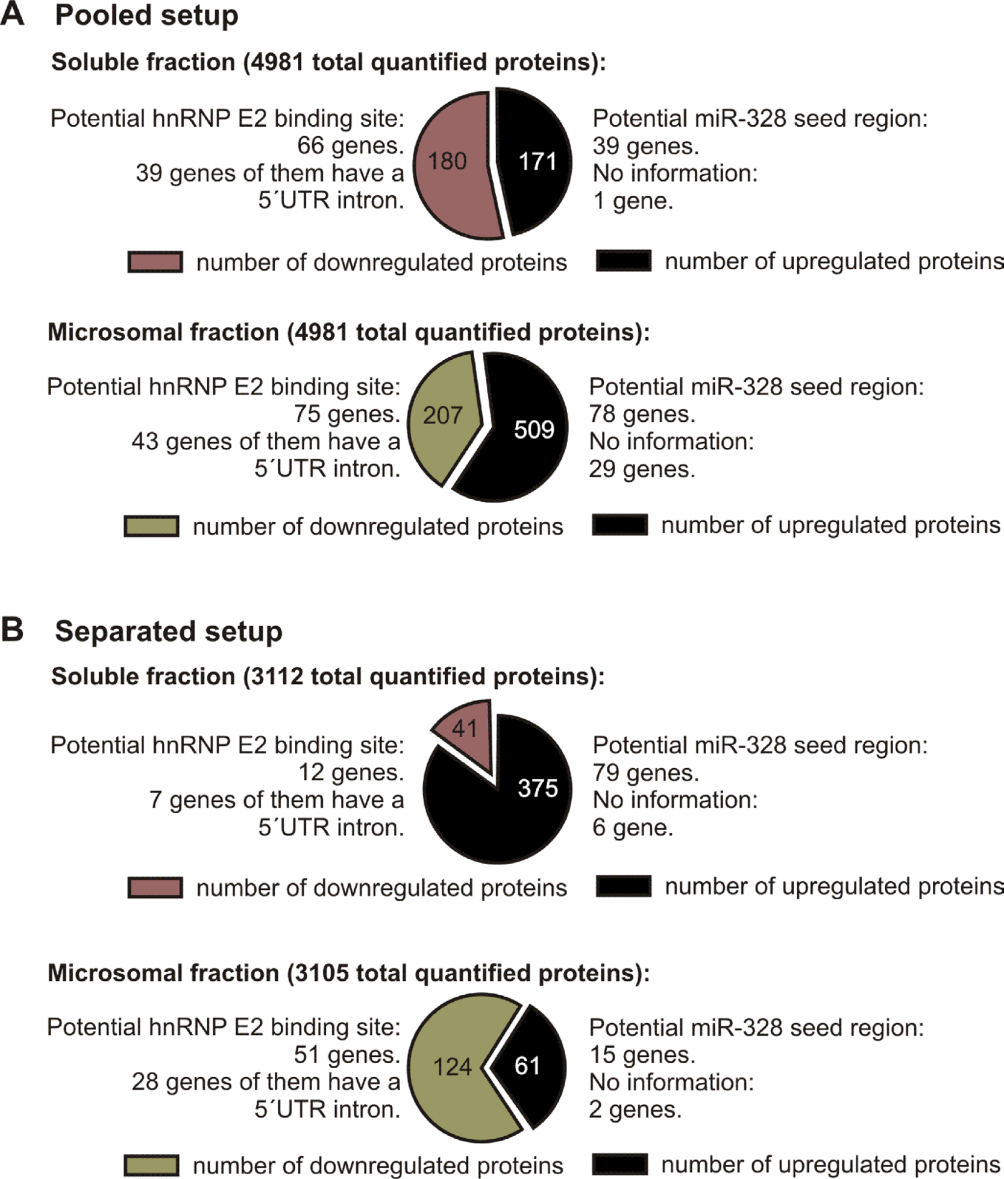
Number of proteins differentially expressed after miR-328 knockdown detected in 4d differentiated MM6 cells (downregulated ≤ −1.3 ratio; upregulated ≥ 1.3 ratio). **(A)** Pooled proteomics setup. **(B)** Separated proteomics setup. Number of total quantified proteins and the number of potential hnRNP E2 and miR-328 targets are indicated above.

To investigate changes in the proteome in response to miR-328 knockdown, a TMT-based proteomics approach was carried out in MM6 cells treated with TGFβ and calcitriol for 4 days (**Figure 1**). Soluble and microsomal fractions were prepared from differentiated MM6 cells as previously described (Eriksson et al., 2008; Saul et al., 2016). After digestion, individual peptide fractions were labeled using TMT 6-plex, which enables a highly sensitive multiplex analysis, is time efficient, and controls for technical variations (Sandberg et al., 2014). TMT quantification was performed by measuring the intensities of fragment reporter ions released from the labels in the tandem MS mode (MS2) during peptide fragmentation. Precursor ions were selected in the full scan mode (MS1) to be fragmented. To identify overall trends in protein expression, we applied two different setups of the TMT-based proteomics approach to detect expression variations of proteins localized in both, microsomal and soluble fraction, and to investigate whether the sample composition influences the outcome of the proteomics study. On the one hand, we pooled all TMT labeled samples and analyzed them by MS, and on the other hand, we analyzed separately TMT-labeled samples of the soluble and microsomal fraction, respectively. In the pooled proteomics setup, we identified and quantified 4,981 proteins in both the soluble and the microsomal fraction, consisting of two or more peptides (confidence level ≥ 99%). In the separated setup, we were able to quantify 3,112 proteins in the soluble fraction and 3,105 proteins in the microsomal fraction with a confidence level ≥ 99%. Overall, we set the criteria for downregulation to a TMT ratio ≤ 0.77 (fold change <−1.3) and upregulation was determined with a TMT ratio of ≥ 1.3 (fold change 1.3). In the pooled proteomics setup, we identified in the soluble fraction 171 upregulated proteins (3.4% of all quantified proteins) and 180 downregulated proteins (3.6% of all quantified proteins) in response to miR-328 knockdown. In the separated setup, 375 of all quantified proteins were increased (12%) and only 41 (1.3% proteins) were downregulated in the soluble fraction upon miR knockdown. Only 2% (61 proteins) of all proteins were upregulated in the microsomal fraction and 4% decreased in response to the miR-328 knockdown (**Figure 2**). A comparison of proteins analyzed in both experimental setups revealed that the majority of the proteins were identified in both proteomics approaches, but to a lower extent in the separated proteomics setup. Moreover, it is noticeable that the expression levels between the two proteomics setups are significantly different. Thus, it is possible that a protein [e.g., high mobility group box 1 (HMGB1)] is noted as downregulated in one proteomics setup, but in the other proteomics setup it is noted as unregulated in response to the miR knockdown (**Figure 2**).

Overall, our results show that on average the miR-328 regulates 5% of all analyzed proteins. Yet the distribution of the miR-328 regulated proteins differ strongly depending on the setup of the MS analytic, which indicates that sample content influences the TMT reporter ion-based proteomics analysis.

### Identification of Novel miR-328 and miR-328/hnRNP E2 Target Genes

Using bioinformatical approaches, we aimed to identify possible novel miR-328 and miR-328/hnRNP E2 decoy targets. As a potential canonical target for miR-328, we determined proteins that were upregulated in response to miR-328 knockdown and revealed a potential seed region within their 3´UTR. We predicted the possible binding sequence for miR-328 using miR target prediction tool “microRNA.org” (http:www.microrna.org) (Betel et al., 2008). A protein that was downregulated by the miR-328 knockdown was determined as a potential noncanonical miR-328 target, if it covers a potential hnRNP E2 binding site within its 5´UTR or 5´UTR intron according to Eiring et al. (2010) and Saul et al. (2016). We downloaded the corresponding 5´UTR sequences of the certain proteins from the database “UCSC Genome Browser” (https://genome.ucsc.edu/) (Kent et al., 2002) and analyzed these by using the online tool “SpliceAid 2” to identify potential hnRNP E2 binding motifs (www.introni.it/spliceaid.html) (Piva et al., 2012).

Although different potential canonical and noncanonical miR-328 targets were identified in the separated as well as in the pooled proteomics setup, the ratios of the potential miR-328 and miR-328/hnRNP E2 target genes were nearly the same. We could show that ∼21% of the upregulated proteins were potential canonical targets of miR-328. Approximately 36% of the downregulated proteins contain a putative hnRNP E2 binding site which represents potential novel, noncanonical targets of miR-328. S100A9, the only confirmed target for miR-328 and hnRNP E2 in MM6 cells (Saul et al., 2016), was detected in our proteomics study as a potential noncanonical miR-328 target, supporting the validity of our proteomics data. In **Tables S1** and **S2** all potential canonical and noncanonical miR-328 targets are listed.

### Pathway Analysis of miR-328 Regulated Proteins

Next, we analyzed all miR-328 regulated proteins in soluble as well as microsomal fraction from both proteomics setups using Ingenuity Pathway Analysis (Ingenuity Systems, www.ingenuity.com). We predicted the five most affected canonical pathways in response to miR-328 knockdown. The canonical pathways with p-values <0.05 were defined as significant. The analysis revealed that the eukaryotic initiation factor 2 (eIF2) signaling, regulation of eukaryotic initiation factor 4 (eIF4) and 70-kDa ribosomal S6 kinase (p70S6K) signaling, mitochondrial dysfunction, protein ubiquitination pathway, and mTOR signaling are the most affected canonical pathways by miR-328 knockdown (**Table 1**). We further analyzed all identified potential canonical and noncanonical miR-328 targets merged from both proteomics by STRING database (Szklarczyk et al., 2017) to create an interacting protein network. Based on this data set, an enrichment of Gene Ontology (GO) analysis was performed to predict which biological processes, cellular compartments, and molecular functions are most affected by miR-328. The results of our GO functional enrichment analysis are listed in **Table S3** for all potential miR-328 targets and in **Table S4** for all noncanonical miR-328 targets. Our results show that both the canonical and the noncanonical miR-328 functions regulate different processes in monocytes in a compartment-specific manner. It should be noted that metabolic processes, e.g., single-organism metabolic process or inflammation-related processes like toll-like receptor 2 (TLR2) signaling pathway, is regulated by the canonical miR-328 function in the soluble fraction. In the microsomal fraction, such enrichment was not observed. Here, we recognized that enzymatic reactions seem to be most affected by the canonical miR function. On the contrary, the noncanonical miR-328 function regulates another spectrum of cellular processes. The formation of extracellular vesicles termed as exosomes, RNA binding functions, and proteins associated with the receptor for advanced glycation end products (RAGE) like HMGB1 and S100A9, seem to be affected by the noncanonical miR-328 function in the microsomal fraction. In the soluble fraction, mRNA processing processes, e.g. mRNA splicing, are mostly affected.

**Table 1:** Five most significantly affected canonical pathways by miR-328 knockdown predicted using IPA (Ingenuity Pathway Analysis software, Ingenuity Systems, www.ingenuity.com). A) Pooled proteomics setup. B) Separated proteomics setup.

Proteomics setup	Top canonical pathways	*p*-value
A) Pooled		
Soluble fraction	eIF2 Signaling	1.33E−48
	Regulation of eIF4 and p70S6K Signaling	1.16E−32
	Mitochondrial Dysfunction	3.19E−28
	Protein Ubiquitination Pathway	2.46E−27
	mTOR Signaling	2.31E−23
Microsomal fraction	eIF2 Signaling	1.33E−48
	Regulation of eIF4 and p70S6K Signaling	1.16E−32
	Mitochondrial Dysfunction	3.19E−28
	Protein Ubiquitination Pathway	2.46E−27
	mTOR Signaling	2.31E−23
B) Separated		
Soluble fraction	eIF2 Signaling	9.79E−56
	Regulation of eIF4 and p70S6K Signaling	1.90E−35
	Protein Ubiquitination Pathway	5.23E−26
	Mitochondrial Dysfunction	1.49E−23
	mTOR Signaling	1.40E−20
Microsomal fraction	eIF2 Signaling	9.79E−56
	Regulation of eIF4 and p70S6K Signaling	2.12E−36
	Mitochondrial Dysfunction	1.84E−35
	Protein Ubiquitination Pathway	1.72E−30
	mTOR Signaling	4.53E−26

### Validation of the Proteomic Data Using Western Blot Analysis

**Figure 3:**
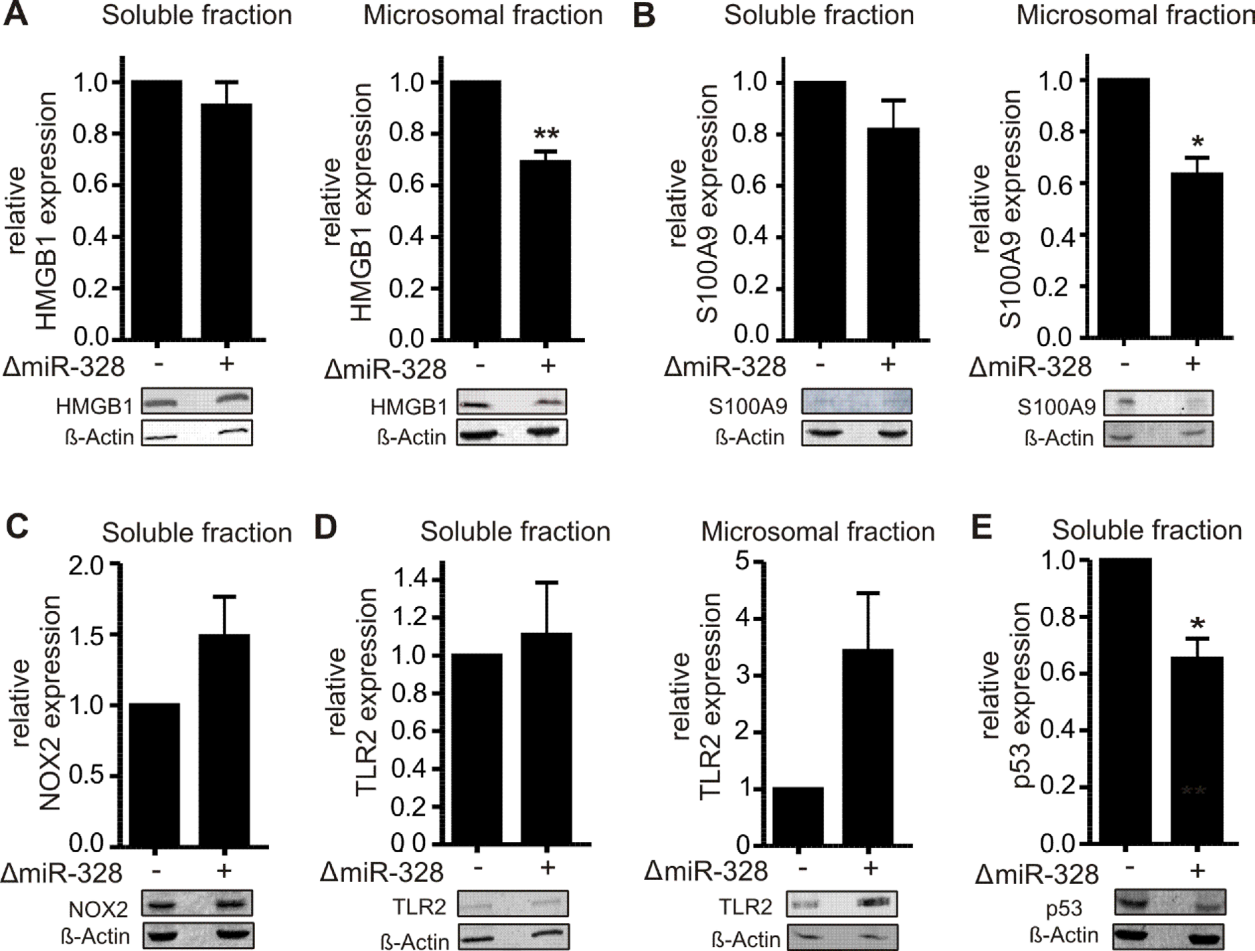
Validation of proteomics results using Western blot analysis of **A)** HMGB1, **B)** S100A9, **C)** NOX2, **D)** TLR2, and **E)** p53 in differentiated MM6 cells with and without knockdown of miR-328 (ΔmiR-328). β-Actin was used as the loading control. The relative changes in ΔmiR-328 samples to control were set as 1 and given as mean + SEM of three independent experiments, except S100A9 expression was analyzed in soluble fraction in four independent experiments, *p < 0.05, **p < 0.01.

In order to validate the proteomics results, different potential canonical and noncanonical miR-328 targets of biological interest were selected to be subjected to Western blot analysis. As potential noncanonical miR-328 target, we have chosen HMGB1, which was downregulated in microsomal fraction by miR-328 knockdown and harbors a potential hnRNP E2 binding site, like S100A9, the known hnRNP E2/miR-328 target (Saul et al., 2016). Both proteins were analyzed by Western blot (**Figure 3A and B**). We would like to note that the expression level of S100A9 in soluble fraction was very low (**Figure 3B**), but overall, the results validated the proteomics data of the pooled setup (**Table S2**).

**Figure 4:**
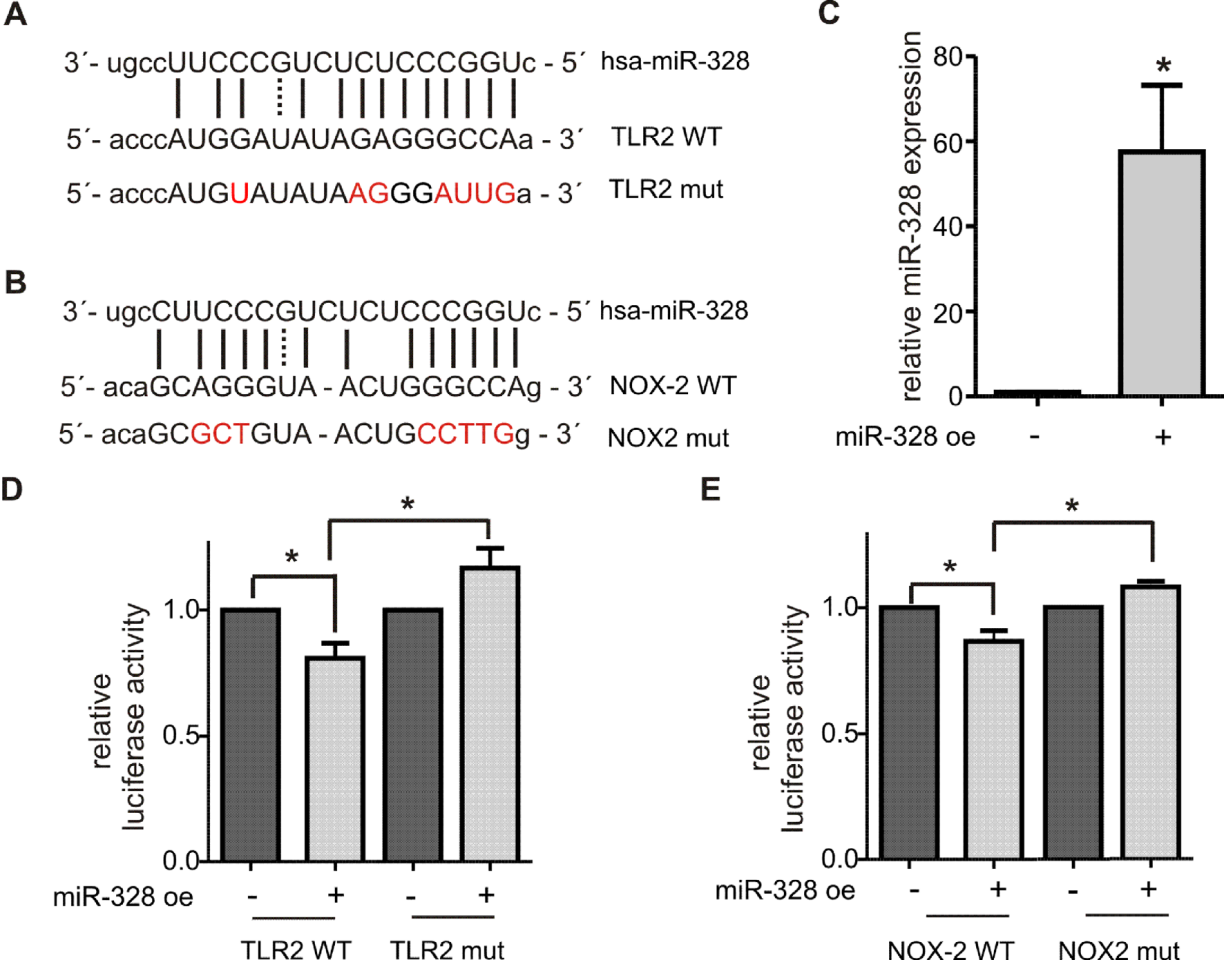
Validation of TLR2 and NOX2 as canonical miR-328 target. Putative miR-328 binding site within the 3´UTR of **(A)** TLR2 wild type (WT) and **(B)** NOX2 WT. Nucleotides mutated for the luciferase reporter gene assays are marked in red. **(C)** Quantification of miR-328 overexpression (miR-328 oe) in HeLa cells using qPCR. Activity of luciferase reporter gene constructs containing **(D)** TLR2 WT or TLR2 WT 3´UTR and **(E)** NOX2 WT or NOX2 mut 3´UTR. The relative luciferase activities are normalized to corresponding oe control. Data are shown as the mean + SEM of minimum three independent experiments. *p < 0.05.

As potential canonical miR-328 targets, we selected NADPH oxidase 2 (NOX2, CYBB) and TLR2. NOX2 and TLR2 were both upregulated in the soluble fraction in response to miR-328 knockdown (**Table S1**). Furthermore, both genes contain putative binding sites for miR-328 within their 3´UTR (**Figure 4A and B**). The Western blot analysis of NOX2 validated our proteomics results in the soluble fraction. Again, NOX2 expression was upregulated in response to miR-328 knockdown (**Figure 3C**). However, the sensitivity of the Western blot analysis was too low to detect any NOX2 expression in the microsomal fraction. Furthermore, we also validated TLR2 as a potential canonical miR-328 target. Analysis was done in both the soluble and microsomal fraction, in which TLR2 expression was strongly upregulated in response to miR-328 knockdown. Of note, we found TLR2 mainly expressed in microsomal, but less in the soluble fraction (**Figure 3D**). We further performed Western blot analysis of tumor protein p53 (p53) **Figure 3E**, since it was predicted as one of the top upstream regulators inhibited upon miR-328 knockdown as shown by Ingenuity Pathway Analysis. Bioinformatical analysis revealed a potential binding site for hnRNP E2 within its 5´UTR intron, similar to S100A9 and HMGB1 which indicates that p53 could represent a novel miR-328/hnRNP E2 target. As predicted, we detected a significant downregulation of p53 expression in response to miR-328 knockdown in the soluble fraction of differentiated MM6 cells which supports our hypothesis that p53 could be a new noncanonical miR-328 target gene. Overall, our Western blot results demonstrate that the expression of the selected proteins were consistent with our TMT-based quantitative proteomics study which confirms the accuracy of our data.

### Validation of Novel Canonical miR-328 Targets

TLR2 and NOX2 were selected for further validations as novel canonical miR-328 targets. The 3´UTR of each gene, harboring a potential seed region of miR-328 (**Figure 4A and B**), was amplified by PCR and cloned downstream of the luciferase open reading frame in the pDLAAG vector (Kemmerer and Weigand, 2014). The constructs were cotransfected with miR-328 mimics into HeLa cells. The overexpression was previously monitored by qPCR and revealed an approximately 60-fold upregulation (**Figure 4C**). The luciferase activity was significantly reduced by both constructs in response to miR-328 overexpression (**Figure 4D and E**). When the potential miR-328 binding sites were mutated (**Figure 4A and B**), the effect of miR-328 knockdown was abolished (**Figure 4D and E**). Overall, our results indicate that TLR2 and NOX2 are direct targets of miR-328.

## Discussion

Numerous publications have shown that miRs are one of the most important posttranscriptional gene expression regulators involved in a variety of biological and physiological processes. The general understanding is that miRs bind sequences related to their target mRNA and mediate either translational repression or degradation of the mRNA transcript (Croce, 2009; Ochs et al., 2011; Ochs et al., 2014). In the last few years, other miR functions have been discovered that lead to an activation of gene expression, e.g. by antagonizing RNA-binding protein activities (Eiring et al., 2010; Saul et al., 2016) or by binding to receptors such as TLR 7/8 (Fabbri et al., 2012). Of note, only a handful of publications describe such new noncanonical functions. This stands in sharp contrast to the thousands of publications that describe the canonical function of miRs. This discrepancy could be explained by the various online prediction tools which makes it rather easy to find a binding site within a 3´UTR and thus a new canonical miR target. Nonetheless, this should not obscure the importance of these noncanonical miR functions. Rather, it should be an incentive to find new miR–protein interactions and to investigate their global significance more closely.

In order to characterize the global role of the noncanonical miR-328 function, we conducted a stable isotope label-based proteomics study to identify new canonical and noncanonical target genes of miR-328 in differentiated MM6 cells and correlate them to their biological function. Subcellular fractionation was applied for comparison of proteins in soluble and microsomal compartment (Ochs et al., 2013). Based on our previous studies, we know that the decoy mechanism occurs mainly in the microsomal fraction (Saul et al., 2016) which indicates that the translational efficiency is modulated mainly on the ER (Ochs et al., 2013). Furthermore, we used two different proteomics setups to span technical variations. Overall, the knockdown of miR-328 leads to a variety of changes in the expression rate of different proteins. Using bioinformatic approaches, we were able to identify proteins that are directly regulated by miR-328 and miR-328/hnRNP E2, respectively, and exclude proteins that were indirectly regulated. Thus, we were able to identify and validate NOX2 and TLR2 as new direct miR-328 targets. It is noteworthy that only about 1/5 of the proteins upregulated by the knockdown have a potential seed sequence for miR-328. This suggests that miR-328 might influence protein expression in other noncanonically ways. In fact, a high number of downregulated proteins in response to miR-328 knockdown contain a potential hnRNP E2 binding site within their 5´UTR or 5´UTR intron which indicates that these proteins are regulated by the noncanonical miR-328 function. We identified for example HMGB1 and p53 as novel potential miR-328/hnRNP E2 targets which needs to be further confirmed in the future. But it is not only the number of regulated proteins that indicate that the noncanonical function of miR-328 has a greater global impact than initially expected. First pathway analysis supports this impression. They demonstrate that both potential miR functions regulate cellular processes in a compartment-specific manner. It should be emphasized that in the microsomal fraction, the extracellular vesicle formation and RAGE signaling are influenced, while in the soluble fraction, mRNA splicing is regulated by the noncanonical miR-328 function. This stands in contrast to the canonical function which affects mostly inflammatory and single organism metabolic processes in the soluble fraction. Of note, specific mechanistic investigations need to be done in order to validate the individual target genes, which were predicted in our proteomics approach.

Overall, our results demonstrate that the canonical and noncanonical miR-functions specifically regulate different cellular processes. It will be of interest in the future to find out how the balance between canonical and noncanonical miR-328 functions is regulated. Our data suggest that the physiological significance of the noncanonical function of the miR-328 is comparable to that of the known canonical miR-function.

## Author Contributions

MS performed the experiments, analyzed the data, conceived the study, designed the project, and wrote the manuscript. EO, IB, KK, and JL performed the MS analytic. AH and MV cloned the vectors. AE performed Western blot experiments. PJ contributed to writing and editing the manuscript. PJ and DS designed the project.

## Funding

This project was supported by the Else Kröner-Fresenius Stiftung (grant no. 2013_A265 and Else Kröner-Fresenius-Graduiertenkolleg), the Deutsche Forschungsgemeinschaft (ECCPS), Athene Young Investigator program (Technische Universität Darmstadt; grant no: n/a), Dr. Ing. Wilhelm und Maria Kirmser-Stiftung (grant no: n/a), Swedish Research Council (grant no. 2017-02577), Innovative Medicines Initiative (EU/EFPIA) (ULTRA-DD grant no. 115766), Stockholm County Council (ALF grant no. 20160378), The Swedish Rheumatism Association (grant no. R-755861), Cancerfonden (grant no. CAN 2016/739), Radiumhemmets forskningsfonder (grant no. 151132), King Gustaf V’s 80-year foundation (grant no. n/a), and funds from Karolinska Institutet (grant no. n/a).

## Conflict of Interest Statement

The authors declare that the research was conducted in the absence of any commercial or financial relationships that could be construed as a potential conflict of interest.
