# Supplementary material for: Mass Spectrometry-Based Proteomics Approach Characterizes the Dual Functionality of miR-328 in Monocytes
Source: Front Pharmacol. 2019 Jun 5;10:640. doi: 10.3389/fphar.2019.00640 (PMC6561313; doi:10.3389/fphar.2019.00640)
Supplement: Supplementary file 1 [file Table_1.docx]

**Mass spectrometry-based proteomics approach characterizes the dual functionality of miR-328 in monocytes**

Meike J. Saul^1,2^*^,^ Anett B. Hegewald^1^, Anne C. Emmerich^1,2^, Elena Ossipova^3^, Marc Vogel^1^, Isabell Baumann^1,2^, Kim Kultima^4^, Johan Lengqivst^3^, Dieter Steinhilber^2^, Per Johan Jakobsson^3^

^1^ Department of Biology, Technische Universität Darmstadt, 64287 Darmstadt, Germany

^2^ Institute of Pharmaceutical Chemistry, Goethe Universität Frankfurt, 60438 Frankfurt/M., Germany

^3^ Rheumatology Unit, Department of Medicine, Solna, Karolinska Institutet, Karolinska University Hospital in Solna, SE- 17176 Stockholm, Sweden

^4^ Department of Medical Sciences, Clinical Chemistry, Uppsala University, SE- 751 85 Uppsala, Sweden

* Correspondence author: Meike J. Saul (saul@bio.tu-darmstadt.de)

Running title: Dual miR-328 functionality in inflammation

Keywords: miR-328, non-canonical function, proteomics, TLR2, NOX2, p53, inflammation

**Table S1. Potential miR-328 targets detected in TMT-based proteomics study** (pooled setup: A-B). A) soluble fraction and B) microsomal fraction. Potential miR-328 targets detected in TMT-based proteomics study (separated setup: C-D). C) soluble fraction and D) microsomal fraction.

| **A) Pooled Proteomics setup: Soluble fraction**  **Potential miR-328 targets** | | |
| --- | --- | --- |
| **Accession** | **Description** | **TMT ratio** |
| Q9H0R8 | Gamma-aminobutyric acid receptor-associated protein-like 1 OS=Homo sapiens GN=GABARAPL1 PE=1 SV=1 - [GBRL1_HUMAN] | 1,90 |
| Q96RL1-3 | Isoform 3 of BRCA1-A complex subunit RAP80 OS=Homo sapiens GN=UIMC1 - [UIMC1_HUMAN] | 1,62 |
| Q8N5Y8 | Mono [ADP-ribose] polymerase PARP16 OS=Homo sapiens GN=PARP16 PE=1 SV=2 - [PAR16_HUMAN] | 1,62 |
| Q9NQC1-3 | Isoform 3 of Protein Jade-2 OS=Homo sapiens GN=JADE2 - [JADE2_HUMAN] | 1,61 |
| Q15041-3 | Isoform 3 of ADP-ribosylation factor-like protein 6-interacting protein 1 OS=Homo sapiens GN=ARL6IP1 - [AR6P1_HUMAN] | 1,53 |
| Q96H55-3 | Isoform 3 of Unconventional myosin-XIX OS=Homo sapiens GN=MYO19 - [MYO19_HUMAN] | 1,52 |
| P08253-2 | Isoform 2 of 72 kDa type IV collagenase OS=Homo sapiens GN=MMP2 - [MMP2_HUMAN] | 1,50 |
| Q9BW60-2 | Isoform 2 of Elongation of very long chain fatty acids protein 1 OS=Homo sapiens GN=ELOVL1 - [ELOV1_HUMAN] | 1,48 |
| P12694 | 2-oxoisovalerate dehydrogenase subunit alpha, mitochondrial OS=Homo sapiens GN=BCKDHA PE=1 SV=2 - [ODBA_HUMAN] | 1,46 |
| Q86TM6-2 | Isoform 2 of E3 ubiquitin-protein ligase synoviolin OS=Homo sapiens GN=SYVN1 - [SYVN1_HUMAN] | 1,45 |
| Q7Z3U7-2 | Isoform 2 of Protein MON2 homolog OS=Homo sapiens GN=MON2 - [MON2_HUMAN] | 1,44 |
| O15533-2 | Isoform 2 of Tapasin OS=Homo sapiens GN=TAPBP - [TPSN_HUMAN] | 1,42 |
| Q12857-2 | Isoform 2 of Nuclear factor 1 A-type OS=Homo sapiens GN=NFIA - [NFIA_HUMAN] | 1,40 |
| Q15363 | Transmembrane emp24 domain-containing protein 2 OS=Homo sapiens GN=TMED2 PE=1 SV=1 - [TMED2_HUMAN] | 1,39 |
| O95674 | Phosphatidate cytidylyltransferase 2 OS=Homo sapiens GN=CDS2 PE=1 SV=1 - [CDS2_HUMAN] | 1,39 |
| Q9NSU2-2 | Isoform 2 of Three-prime repair exonuclease 1 OS=Homo sapiens GN=TREX1 - [TREX1_HUMAN] | 1,39 |
| Q96NT0 | Coiled-coil domain-containing protein 115 OS=Homo sapiens GN=CCDC115 PE=1 SV=1 - [CC115_HUMAN] | 1,38 |
| O14735-3 | Isoform 3 of CDP-diacylglycerol--inositol 3-phosphatidyltransferase OS=Homo sapiens GN=CDIPT - [CDIPT_HUMAN] | 1,36 |
| Q9H936 | Mitochondrial glutamate carrier 1 OS=Homo sapiens GN=SLC25A22 PE=1 SV=1 - [GHC1_HUMAN] | 1,35 |
| Q15392-2 | Isoform 2 of Delta(24)-sterol reductase OS=Homo sapiens GN=DHCR24 - [DHC24_HUMAN] | 1,34 |
| Q5BJF2 | Transmembrane protein 97 OS=Homo sapiens GN=TMEM97 PE=1 SV=1 - [TMM97_HUMAN] | 1,34 |
| Q9Y487 | V-type proton ATPase 116 kDa subunit a isoform 2 OS=Homo sapiens GN=ATP6V0A2 PE=1 SV=2 - [VPP2_HUMAN] | 1,33 |
| Q9BQA9-2 | Isoform 2 of Uncharacterized protein C17orf62 OS=Homo sapiens GN=C17orf62 - [CQ062_HUMAN] | 1,32 |
| Q96GQ5 | RUS1 family protein C16orf58 OS=Homo sapiens GN=C16orf58 PE=1 SV=2 - [RUS1_HUMAN] | 1,32 |
| Q96G23 | Ceramide synthase 2 OS=Homo sapiens GN=CERS2 PE=1 SV=1 - [CERS2_HUMAN] | 1,32 |
| P46977 | Dolichyl-diphosphooligosaccharide--protein glycosyltransferase subunit STT3A OS=Homo sapiens GN=STT3A PE=1 SV=2 - [STT3A_HUMAN] | 1,32 |
| Q15005 | Signal peptidase complex subunit 2 OS=Homo sapiens GN=SPCS2 PE=1 SV=3 - [SPCS2_HUMAN] | 1,32 |
| Q969J3-2 | Isoform 2 of Loss of heterozygosity 12 chromosomal region 1 protein OS=Homo sapiens GN=LOH12CR1 - [L12R1_HUMAN] | 1,32 |
| Q9NRA2 | Sialin OS=Homo sapiens GN=SLC17A5 PE=1 SV=2 - [S17A5_HUMAN] | 1,32 |
| Q9UNL2 | Translocon-associated protein subunit gamma OS=Homo sapiens GN=SSR3 PE=1 SV=1 - [SSRG_HUMAN] | 1,31 |
| Q8NBI5 | Solute carrier family 43 member 3 OS=Homo sapiens GN=SLC43A3 PE=1 SV=2 - [S43A3_HUMAN] | 1,31 |
| P43307 | Translocon-associated protein subunit alpha OS=Homo sapiens GN=SSR1 PE=1 SV=3 - [SSRA_HUMAN] | 1,31 |
| Q58EX2-2 | Isoform 2 of Protein sidekick-2 OS=Homo sapiens GN=SDK2 - [SDK2_HUMAN] | 1,31 |
| Q5T9A4 | ATPase family AAA domain-containing protein 3B OS=Homo sapiens GN=ATAD3B PE=1 SV=1 - [ATD3B_HUMAN] | 1,31 |
| Q2TAA5 | GDP-Man:Man(3)GlcNAc(2)-PP-Dol alpha-1,2-mannosyltransferase OS=Homo sapiens GN=ALG11 PE=1 SV=2 - [ALG11_HUMAN] | 1,31 |
| Q12770 | Sterol regulatory element-binding protein cleavage-activating protein OS=Homo sapiens GN=SCAP PE=1 SV=4 - [SCAP_HUMAN] | 1,30 |
| P51798-2 | Isoform 2 of H(+)/Cl(-) exchange transporter 7 OS=Homo sapiens GN=CLCN7 - [CLCN7_HUMAN] | 1,30 |
| Q86UL3 | Glycerol-3-phosphate acyltransferase 4 OS=Homo sapiens GN=AGPAT6 PE=1 SV=1 - [GPAT4_HUMAN] | 1,30 |
| Q8TCT9-5 | Isoform 5 of Minor histocompatibility antigen H13 OS=Homo sapiens GN=HM13 - [HM13_HUMAN] | 1,30 |

| **B) Pooled Proteomics setup: Microsomal fraction**  **Potential miR-328 targets** | | |
| --- | --- | --- |
| **Accession** | **Description** | **TMT ratio** |
| Q86YR5-3 | Isoform 3 of G-protein-signaling modulator 1 OS=Homo sapiens GN=GPSM1 - [GPSM1_HUMAN] | 2,61 |
| Q9BVC4-3 | Isoform 2 of Target of rapamycin complex subunit LST8 OS=Homo sapiens GN=MLST8 - [LST8_HUMAN] | 2,46 |
| P42684-8 | Isoform 8 of Abelson tyrosine-protein kinase 2 OS=Homo sapiens GN=ABL2 - [ABL2_HUMAN] | 2,45 |
| Q8IY37 | Probable ATP-dependent RNA helicase DHX37 OS=Homo sapiens GN=DHX37 PE=1 SV=1 - [DHX37_HUMAN] | 2,28 |
| Q9NQ66-2 | Isoform B of 1-phosphatidylinositol 4,5-bisphosphate phosphodiesterase beta-1 OS=Homo sapiens GN=PLCB1 - [PLCB1_HUMAN] | 2,21 |
| Q8NI60-2 | Isoform 2 of Atypical kinase ADCK3, mitochondrial OS=Homo sapiens GN=ADCK3 - [ADCK3_HUMAN] | 2,16 |
| P49336-2 | Isoform 2 of Cyclin-dependent kinase 8 OS=Homo sapiens GN=CDK8 - [CDK8_HUMAN] | 2,08 |
| Q86YT6 | E3 ubiquitin-protein ligase MIB1 OS=Homo sapiens GN=MIB1 PE=1 SV=1 - [MIB1_HUMAN] | 2,03 |
| P51812 | Ribosomal protein S6 kinase alpha-3 OS=Homo sapiens GN=RPS6KA3 PE=1 SV=1 - [KS6A3_HUMAN] | 1,73 |
| O14787-2 | Isoform 2 of Transportin-2 OS=Homo sapiens GN=TNPO2 - [TNPO2_HUMAN] | 1,70 |
| Q92551-2 | Isoform 2 of Inositol hexakisphosphate kinase 1 OS=Homo sapiens GN=IP6K1 - [IP6K1_HUMAN] | 1,69 |
| Q96AB3-3 | Isoform 3 of Isochorismatase domain-containing protein 2, mitochondrial OS=Homo sapiens GN=ISOC2 - [ISOC2_HUMAN] | 1,68 |
| Q7LGA3 | Heparan sulfate 2-O-sulfotransferase 1 OS=Homo sapiens GN=HS2ST1 PE=1 SV=1 - [HS2ST_HUMAN] | 1,64 |
| O00767 | Acyl-CoA desaturase OS=Homo sapiens GN=SCD PE=1 SV=2 - [ACOD_HUMAN] | 1,64 |
| Q86UX7-2 | Isoform 2 of Fermitin family homolog 3 OS=Homo sapiens GN=FERMT3 - [URP2_HUMAN] | 1,64 |
| Q9NP64-2 | Isoform 2 of Nucleolar protein of 40 kDa OS=Homo sapiens GN=ZCCHC17 - [NO40_HUMAN] | 1,64 |
| Q5BJF2 | Transmembrane protein 97 OS=Homo sapiens GN=TMEM97 PE=1 SV=1 - [TMM97_HUMAN] | 1,61 |
| Q00325-2 | Isoform B of Phosphate carrier protein, mitochondrial OS=Homo sapiens GN=SLC25A3 - [MPCP_HUMAN] | 1,61 |
| P49795 | Regulator of G-protein signaling 19 OS=Homo sapiens GN=RGS19 PE=1 SV=1 - [RGS19_HUMAN] | 1,59 |
| Q96P70 | Importin-9 OS=Homo sapiens GN=IPO9 PE=1 SV=3 - [IPO9_HUMAN] | 1,56 |
| Q9BQA9-2 | Isoform 2 of Uncharacterized protein C17orf62 OS=Homo sapiens GN=C17orf62 - [CQ062_HUMAN] | 1,56 |
| Q07817-2 | Isoform Bcl-X(S) of Bcl-2-like protein 1 OS=Homo sapiens GN=BCL2L1 - [B2CL1_HUMAN] | 1,55 |
| Q96CX6 | Leucine-rich repeat-containing protein 58 OS=Homo sapiens GN=LRRC58 PE=1 SV=2 - [LRC58_HUMAN] | 1,55 |
| Q16584 | Mitogen-activated protein kinase kinase kinase 11 OS=Homo sapiens GN=MAP3K11 PE=1 SV=1 - [M3K11_HUMAN] | 1,55 |
| Q7Z3U7-2 | Isoform 2 of Protein MON2 homolog OS=Homo sapiens GN=MON2 - [MON2_HUMAN] | 1,54 |
| Q7LDG7-3 | Isoform 3 of RAS guanyl-releasing protein 2 OS=Homo sapiens GN=RASGRP2 - [GRP2_HUMAN] | 1,54 |
| Q12931-2 | Isoform 2 of Heat shock protein 75 kDa, mitochondrial OS=Homo sapiens GN=TRAP1 - [TRAP1_HUMAN] | 1,53 |
| Q07960 | Rho GTPase-activating protein 1 OS=Homo sapiens GN=ARHGAP1 PE=1 SV=1 - [RHG01_HUMAN] | 1,53 |
| Q92973-2 | Isoform 2 of Transportin-1 OS=Homo sapiens GN=TNPO1 - [TNPO1_HUMAN] | 1,53 |
| Q5QJ74 | Tubulin-specific chaperone cofactor E-like protein OS=Homo sapiens GN=TBCEL PE=1 SV=2 - [TBCEL_HUMAN] | 1,53 |
| Q68DK2-3 | Isoform 3 of Zinc finger FYVE domain-containing protein 26 OS=Homo sapiens GN=ZFYVE26 - [ZFY26_HUMAN] | 1,52 |
| Q58EX2-2 | Isoform 2 of Protein sidekick-2 OS=Homo sapiens GN=SDK2 - [SDK2_HUMAN] | 1,52 |
| Q15418-4 | Isoform 4 of Ribosomal protein S6 kinase alpha-1 OS=Homo sapiens GN=RPS6KA1 - [KS6A1_HUMAN] | 1,52 |
| O94927 | HAUS augmin-like complex subunit 5 OS=Homo sapiens GN=HAUS5 PE=1 SV=2 - [HAUS5_HUMAN] | 1,52 |
| O43414-3 | Isoform 3 of ERI1 exoribonuclease 3 OS=Homo sapiens GN=ERI3 - [ERI3_HUMAN] | 1,51 |
| Q9NQT8 | Kinesin-like protein KIF13B OS=Homo sapiens GN=KIF13B PE=1 SV=2 - [KI13B_HUMAN] | 1,50 |
| Q9NWZ3 | Interleukin-1 receptor-associated kinase 4 OS=Homo sapiens GN=IRAK4 PE=1 SV=1 - [IRAK4_HUMAN] | 1,50 |
| Q9Y6Y0 | Influenza virus NS1A-binding protein OS=Homo sapiens GN=IVNS1ABP PE=1 SV=3 - [NS1BP_HUMAN] | 1,49 |
| Q8TCT9-5 | Isoform 5 of Minor histocompatibility antigen H13 OS=Homo sapiens GN=HM13 - [HM13_HUMAN] | 1,49 |
| P62745 | Rho-related GTP-binding protein RhoB OS=Homo sapiens GN=RHOB PE=1 SV=1 - [RHOB_HUMAN] | 1,49 |
| Q9BW60-2 | Isoform 2 of Elongation of very long chain fatty acids protein 1 OS=Homo sapiens GN=ELOVL1 - [ELOV1_HUMAN] | 1,49 |
| Q9NZN4 | EH domain-containing protein 2 OS=Homo sapiens GN=EHD2 PE=1 SV=2 - [EHD2_HUMAN] | 1,48 |
| P40937 | Replication factor C subunit 5 OS=Homo sapiens GN=RFC5 PE=1 SV=1 - [RFC5_HUMAN] | 1,48 |
| P35226 | Polycomb complex protein BMI-1 OS=Homo sapiens GN=BMI1 PE=1 SV=2 - [BMI1_HUMAN] | 1,48 |
| Q9H6D7-3 | Isoform 3 of HAUS augmin-like complex subunit 4 OS=Homo sapiens GN=HAUS4 - [HAUS4_HUMAN] | 1,47 |
| P21266 | Glutathione S-transferase Mu 3 OS=Homo sapiens GN=GSTM3 PE=1 SV=3 - [GSTM3_HUMAN] | 1,47 |
| Q96JH7 | Deubiquitinating protein VCIP135 OS=Homo sapiens GN=VCPIP1 PE=1 SV=2 - [VCIP1_HUMAN] | 1,46 |
| Q9H936 | Mitochondrial glutamate carrier 1 OS=Homo sapiens GN=SLC25A22 PE=1 SV=1 - [GHC1_HUMAN] | 1,46 |
| Q9Y5S2 | Serine/threonine-protein kinase MRCK beta OS=Homo sapiens GN=CDC42BPB PE=1 SV=2 - [MRCKB_HUMAN] | 1,45 |
| Q684P5-3 | Isoform 3 of Rap1 GTPase-activating protein 2 OS=Homo sapiens GN=RAP1GAP2 - [RPGP2_HUMAN] | 1,45 |
| Q9NZJ7-2 | Isoform 2 of Mitochondrial carrier homolog 1 OS=Homo sapiens GN=MTCH1 - [MTCH1_HUMAN] | 1,45 |
| P19388 | DNA-directed RNA polymerases I, II, and III subunit RPABC1 OS=Homo sapiens GN=POLR2E PE=1 SV=4 - [RPAB1_HUMAN] | 1,43 |
| Q9H939-2 | Isoform 2 of Proline-serine-threonine phosphatase-interacting protein 2 OS=Homo sapiens GN=PSTPIP2 - [PPIP2_HUMAN] | 1,43 |
| Q8N4Q0 | Zinc-binding alcohol dehydrogenase domain-containing protein 2 OS=Homo sapiens GN=ZADH2 PE=1 SV=1 - [ZADH2_HUMAN] | 1,42 |
| Q9Y5K5-2 | Isoform 2 of Ubiquitin carboxyl-terminal hydrolase isozyme L5 OS=Homo sapiens GN=UCHL5 - [UCHL5_HUMAN] | 1,42 |
| Q15363 | Transmembrane emp24 domain-containing protein 2 OS=Homo sapiens GN=TMED2 PE=1 SV=1 - [TMED2_HUMAN] | 1,42 |
| Q15528-2 | Isoform Surf5A of Mediator of RNA polymerase II transcription subunit 22 OS=Homo sapiens GN=MED22 - [MED22_HUMAN] | 1,40 |
| Q5T9A4 | ATPase family AAA domain-containing protein 3B OS=Homo sapiens GN=ATAD3B PE=1 SV=1 - [ATD3B_HUMAN] | 1,38 |
| Q9NZI7-4 | Isoform 2 of Upstream-binding protein 1 OS=Homo sapiens GN=UBP1 - [UBIP1_HUMAN] | 1,37 |
| Q9NUM3-3 | Isoform 3 of Zinc transporter ZIP9 OS=Homo sapiens GN=SLC39A9 - [S39A9_HUMAN] | 1,37 |
| Q12913-2 | Isoform 2 of Receptor-type tyrosine-protein phosphatase eta OS=Homo sapiens GN=PTPRJ - [PTPRJ_HUMAN] | 1,37 |
| Q8N5M9 | Protein jagunal homolog 1 OS=Homo sapiens GN=JAGN1 PE=1 SV=1 - [JAGN1_HUMAN] | 1,36 |
| Q8WZA9 | Immunity-related GTPase family Q protein OS=Homo sapiens GN=IRGQ PE=1 SV=1 - [IRGQ_HUMAN] | 1,36 |
| P16615 | Sarcoplasmic/endoplasmic reticulum calcium ATPase 2 OS=Homo sapiens GN=ATP2A2 PE=1 SV=1 - [AT2A2_HUMAN] | 1,36 |
| Q8TCE6-2 | Isoform 2 of Protein FAM45A OS=Homo sapiens GN=FAM45A - [FA45A_HUMAN] | 1,36 |
| Q9NUQ8-2 | Isoform 2 of ATP-binding cassette sub-family F member 3 OS=Homo sapiens GN=ABCF3 - [ABCF3_HUMAN] | 1,36 |
| Q9BV68-2 | Isoform 2 of E3 ubiquitin-protein ligase RNF126 OS=Homo sapiens GN=RNF126 - [RN126_HUMAN] | 1,36 |
| Q6PI78 | Transmembrane protein 65 OS=Homo sapiens GN=TMEM65 PE=1 SV=2 - [TMM65_HUMAN] | 1,35 |
| Q8IXT5 | RNA-binding protein 12B OS=Homo sapiens GN=RBM12B PE=1 SV=2 - [RB12B_HUMAN] | 1,34 |
| Q99460-2 | Isoform 2 of 26S proteasome non-ATPase regulatory subunit 1 OS=Homo sapiens GN=PSMD1 - [PSMD1_HUMAN] | 1,34 |
| P50416-2 | Isoform 2 of Carnitine O-palmitoyltransferase 1, liver isoform OS=Homo sapiens GN=CPT1A - [CPT1A_HUMAN] | 1,33 |
| P10586-2 | Isoform 2 of Receptor-type tyrosine-protein phosphatase F OS=Homo sapiens GN=PTPRF - [PTPRF_HUMAN] | 1,31 |
| Q9UGN4-4 | Isoform 4 of CMRF35-like molecule 8 OS=Homo sapiens GN=CD300A - [CLM8_HUMAN] | 1,31 |
| Q5JTZ9 | Alanine--tRNA ligase, mitochondrial OS=Homo sapiens GN=AARS2 PE=1 SV=1 - [SYAM_HUMAN] | 1,30 |
| P30519 | Heme oxygenase 2 OS=Homo sapiens GN=HMOX2 PE=1 SV=2 - [HMOX2_HUMAN] | 1,30 |
| Q99873-3 | Isoform 3 of Protein arginine N-methyltransferase 1 OS=Homo sapiens GN=PRMT1 - [ANM1_HUMAN] | 1,30 |
| Q9Y4P1-4 | Isoform 4 of Cysteine protease ATG4B OS=Homo sapiens GN=ATG4B - [ATG4B_HUMAN] | 1,30 |
| Q9UHX1-4 | Isoform 4 of Poly(U)-binding-splicing factor PUF60 OS=Homo sapiens GN=PUF60 - [PUF60_HUMAN] | 1,30 |

| **C) Separated Proteomics setup: Soluble fraction**  **Potential miR-328 targets** | | |
| --- | --- | --- |
| **Accession** | **Description** | **TMT ratio** |
| E9PL01 | Signal peptidase complex subunit 2 OS=Homo sapiens GN=SPCS2 PE=1 SV=1 - [E9PL01_HUMAN] | 3,42 |
| Q8TCD5-2 | Isoform 2 of 5'(3')-deoxyribonucleotidase, cytosolic type OS=Homo sapiens GN=NT5C - [NT5C_HUMAN] | 2,98 |
| Q8TCT9-5 | Isoform 5 of Minor histocompatibility antigen H13 OS=Homo sapiens GN=HM13 - [HM13_HUMAN] | 2,30 |
| F5GX30 | Cation-dependent mannose-6-phosphate receptor OS=Homo sapiens GN=M6PR PE=1 SV=2 - [F5GX30_HUMAN] | 2,09 |
| F5H8H2 | Mevalonate kinase OS=Homo sapiens GN=MVK PE=1 SV=1 - [F5H8H2_HUMAN] | 1,90 |
| B7Z2L0 | B-cell receptor-associated protein 29 OS=Homo sapiens GN=BCAP29 PE=1 SV=1 - [B7Z2L0_HUMAN] | 1,86 |
| P46977-2 | Isoform 2 of Dolichyl-diphosphooligosaccharide--protein glycosyltransferase subunit STT3A OS=Homo sapiens GN=STT3A - [STT3A_HUMAN] | 1,85 |
| F5GX39 | Transmembrane emp24 domain-containing protein 2 OS=Homo sapiens GN=TMED2 PE=1 SV=1 - [F5GX39_HUMAN] | 1,80 |
| H7C3K7 | Synaptobrevin homolog YKT6 (Fragment) OS=Homo sapiens GN=YKT6 PE=1 SV=1 - [H7C3K7_HUMAN] | 1,79 |
| P16615-2 | Isoform 2 of Sarcoplasmic/endoplasmic reticulum calcium ATPase 2 OS=Homo sapiens GN=ATP2A2 - [AT2A2_HUMAN] | 1,78 |
| F5H304 | Replication factor C subunit 5 (Fragment) OS=Homo sapiens GN=RFC5 PE=1 SV=1 - [F5H304_HUMAN] | 1,76 |
| Q53GQ0 | Very-long-chain 3-oxoacyl-CoA reductase OS=Homo sapiens GN=HSD17B12 PE=1 SV=2 - [DHB12_HUMAN] | 1,75 |
| C9JA28 | Translocon-associated protein subunit gamma OS=Homo sapiens GN=SSR3 PE=1 SV=1 - [C9JA28_HUMAN] | 1,73 |
| F8W681 | UDP-N-acetylglucosamine--dolichyl-phosphate N-acetylglucosaminephosphotransferase OS=Homo sapiens GN=DPAGT1 PE=1 SV=1 - [F8W681_HUMAN] | 1,68 |
| H7C4J4 | Armadillo repeat-containing protein 8 (Fragment) OS=Homo sapiens GN=ARMC8 PE=1 SV=1 - [H7C4J4_HUMAN] | 1,68 |
| O95373 | Importin-7 OS=Homo sapiens GN=IPO7 PE=1 SV=1 - [IPO7_HUMAN] | 1,68 |
| I3L286 | Ubiquitin-conjugating enzyme E2 Z (Fragment) OS=Homo sapiens GN=UBE2Z PE=1 SV=1 - [I3L286_HUMAN] | 1,67 |
| Q92608 | Dedicator of cytokinesis protein 2 OS=Homo sapiens GN=DOCK2 PE=1 SV=2 - [DOCK2_HUMAN] | 1,66 |
| C9J3L8 | Translocon-associated protein subunit alpha OS=Homo sapiens GN=SSR1 PE=1 SV=1 - [C9J3L8_HUMAN] | 1,66 |
| F5H3Y4 | Probable ATP-dependent RNA helicase DHX37 OS=Homo sapiens GN=DHX37 PE=1 SV=1 - [F5H3Y4_HUMAN] | 1,64 |
| A2IDC7 | 39S ribosomal protein L28, mitochondrial (Fragment) OS=Homo sapiens GN=MRPL28 PE=1 SV=1 - [A2IDC7_HUMAN] | 1,63 |
| Q9UM00 | Transmembrane and coiled-coil domain-containing protein 1 OS=Homo sapiens GN=TMCO1 PE=1 SV=1 - [TMCO1_HUMAN] | 1,62 |
| Q6P1N7 | TAPBP protein OS=Homo sapiens GN=TAPBP PE=1 SV=1 - [Q6P1N7_HUMAN] | 1,61 |
| K7EIL9 | Histone chaperone ASF1B OS=Homo sapiens GN=ASF1B PE=1 SV=1 - [K7EIL9_HUMAN] | 1,60 |
| Q8N5K1 | CDGSH iron-sulfur domain-containing protein 2 OS=Homo sapiens GN=CISD2 PE=1 SV=1 - [CISD2_HUMAN] | 1,60 |
| P21266 | Glutathione S-transferase Mu 3 OS=Homo sapiens GN=GSTM3 PE=1 SV=3 - [GSTM3_HUMAN] | 1,57 |
| Q96HR9 | Receptor expression-enhancing protein 6 OS=Homo sapiens GN=REEP6 PE=1 SV=1 - [REEP6_HUMAN] | 1,57 |
| O00767 | Acyl-CoA desaturase OS=Homo sapiens GN=SCD PE=1 SV=2 - [ACOD_HUMAN] | 1,55 |
| A0A087WY88 | Protein jagunal homolog 1 OS=Homo sapiens GN=JAGN1 PE=1 SV=1 - [A0A087WY88_HUMAN] | 1,55 |
| Q8WUM4 | Programmed cell death 6-interacting protein OS=Homo sapiens GN=PDCD6IP PE=1 SV=1 - [PDC6I_HUMAN] | 1,53 |
| Q92973-2 | Isoform 2 of Transportin-1 OS=Homo sapiens GN=TNPO1 - [TNPO1_HUMAN] | 1,52 |
| Q86TB9-2 | Isoform 2 of Protein PAT1 homolog 1 OS=Homo sapiens GN=PATL1 - [PATL1_HUMAN] | 1,50 |
| Q14738-3 | Isoform Delta-3 of Serine/threonine-protein phosphatase 2A 56 kDa regulatory subunit delta isoform OS=Homo sapiens GN=PPP2R5D - [2A5D_HUMAN] | 1,50 |
| C9J4I3 | Signal transducer and activator of transcription 5B (Fragment) OS=Homo sapiens GN=STAT5B PE=1 SV=1 - [C9J4I3_HUMAN] | 1,49 |
| Q96P70 | Importin-9 OS=Homo sapiens GN=IPO9 PE=1 SV=3 - [IPO9_HUMAN] | 1,49 |
| P04839 | Cytochrome b-245 heavy chain OS=Homo sapiens GN=CYBB PE=1 SV=2 - [CY24B_HUMAN] | 1,47 |
| Q9NWZ3-2 | Isoform 2 of Interleukin-1 receptor-associated kinase 4 OS=Homo sapiens GN=IRAK4 - [IRAK4_HUMAN] | 1,47 |
| P15153 | Ras-related C3 botulinum toxin substrate 2 OS=Homo sapiens GN=RAC2 PE=1 SV=1 - [RAC2_HUMAN] | 1,46 |
| Q13330-2 | Isoform Short of Metastasis-associated protein MTA1 OS=Homo sapiens GN=MTA1 - [MTA1_HUMAN] | 1,46 |
| Q8TEK8 | FLJ00186 protein (Fragment) OS=Homo sapiens GN=FLJ00186 PE=1 SV=1 - [Q8TEK8_HUMAN] | 1,46 |
| B4E0K5 | Mitogen-activated protein kinase 14 OS=Homo sapiens GN=MAPK14 PE=1 SV=1 - [B4E0K5_HUMAN] | 1,45 |
| E5RGR9 | Protein YIPF5 (Fragment) OS=Homo sapiens GN=YIPF5 PE=1 SV=1 - [E5RGR9_HUMAN] | 1,45 |
| Q5JW01 | Engulfment and cell motility protein 2 (Fragment) OS=Homo sapiens GN=ELMO2 PE=1 SV=1 - [Q5JW01_HUMAN] | 1,44 |
| Q5T2T1 | MAGUK p55 subfamily member 7 OS=Homo sapiens GN=MPP7 PE=1 SV=1 - [MPP7_HUMAN] | 1,44 |
| H7C4B7 | Delta(24)-sterol reductase (Fragment) OS=Homo sapiens GN=DHCR24 PE=1 SV=1 - [H7C4B7_HUMAN] | 1,44 |
| Q9UHG3-2 | Isoform 2 of Prenylcysteine oxidase 1 OS=Homo sapiens GN=PCYOX1 - [PCYOX_HUMAN] | 1,43 |
| Q9Y5M8 | Signal recognition particle receptor subunit beta OS=Homo sapiens GN=SRPRB PE=1 SV=3 - [SRPRB_HUMAN] | 1,42 |
| O43615 | Mitochondrial import inner membrane translocase subunit TIM44 OS=Homo sapiens GN=TIMM44 PE=1 SV=2 - [TIM44_HUMAN] | 1,42 |
| P35232 | Prohibitin OS=Homo sapiens GN=PHB PE=1 SV=1 - [PHB_HUMAN] | 1,41 |
| Q13107-3 | Isoform 3 of Ubiquitin carboxyl-terminal hydrolase 4 OS=Homo sapiens GN=USP4 - [UBP4_HUMAN] | 1,40 |
| Q9Y3T9 | Nucleolar complex protein 2 homolog OS=Homo sapiens GN=NOC2L PE=1 SV=4 - [NOC2L_HUMAN] | 1,40 |
| Q07960 | Rho GTPase-activating protein 1 OS=Homo sapiens GN=ARHGAP1 PE=1 SV=1 - [RHG01_HUMAN] | 1,39 |
| K7EKR9 | Ribonucleoprotein PTB-binding 1 (Fragment) OS=Homo sapiens GN=RAVER1 PE=1 SV=1 - [K7EKR9_HUMAN] | 1,37 |
| Q08722-2 | Isoform OA3-293 of Leukocyte surface antigen CD47 OS=Homo sapiens GN=CD47 - [CD47_HUMAN] | 1,37 |
| Q8TEM1 | Nuclear pore membrane glycoprotein 210 OS=Homo sapiens GN=NUP210 PE=1 SV=3 - [PO210_HUMAN] | 1,37 |
| F8WEP8 | Upstream-binding protein 1 OS=Homo sapiens GN=UBP1 PE=1 SV=1 - [F8WEP8_HUMAN] | 1,37 |
| Q96K76-3 | Isoform 3 of Ubiquitin carboxyl-terminal hydrolase 47 OS=Homo sapiens GN=USP47 - [UBP47_HUMAN] | 1,37 |
| K7EK17 | MAP/microtubule affinity-regulating kinase 4 (Fragment) OS=Homo sapiens GN=MARK4 PE=1 SV=1 - [K7EK17_HUMAN] | 1,36 |
| Q9H3N1 | Thioredoxin-related transmembrane protein 1 OS=Homo sapiens GN=TMX1 PE=1 SV=1 - [TMX1_HUMAN] | 1,36 |
| F8WF48 | Translocation protein SEC62 OS=Homo sapiens GN=SEC62 PE=1 SV=1 - [F8WF48_HUMAN] | 1,35 |
| Q15418-4 | Isoform 4 of Ribosomal protein S6 kinase alpha-1 OS=Homo sapiens GN=RPS6KA1 - [KS6A1_HUMAN] | 1,35 |
| P54577 | Tyrosine--tRNA ligase, cytoplasmic OS=Homo sapiens GN=YARS PE=1 SV=4 - [SYYC_HUMAN] | 1,35 |
| C9JP16 | Cartilage-associated protein OS=Homo sapiens GN=CRTAP PE=1 SV=1 - [C9JP16_HUMAN] | 1,34 |
| G3V2G6 | Retinol dehydrogenase 11 (Fragment) OS=Homo sapiens GN=RDH11 PE=1 SV=1 - [G3V2G6_HUMAN] | 1,34 |
| P00387-2 | Isoform 2 of NADH-cytochrome b5 reductase 3 OS=Homo sapiens GN=CYB5R3 - [NB5R3_HUMAN] | 1,34 |
| F5H1S8 | Malectin (Fragment) OS=Homo sapiens GN=MLEC PE=1 SV=1 - [F5H1S8_HUMAN] | 1,34 |
| Q9NUQ8-2 | Isoform 2 of ATP-binding cassette sub-family F member 3 OS=Homo sapiens GN=ABCF3 - [ABCF3_HUMAN] | 1,34 |
| P29350 | Tyrosine-protein phosphatase non-receptor type 6 OS=Homo sapiens GN=PTPN6 PE=1 SV=1 - [PTN6_HUMAN] | 1,33 |
| Q15003-2 | Isoform 2 of Condensin complex subunit 2 OS=Homo sapiens GN=NCAPH - [CND2_HUMAN] | 1,32 |
| Q9BSJ8 | Extended synaptotagmin-1 OS=Homo sapiens GN=ESYT1 PE=1 SV=1 - [ESYT1_HUMAN] | 1,32 |
| F8WEL7 | Cleavage and polyadenylation-specificity factor subunit 4 OS=Homo sapiens GN=CPSF4 PE=1 SV=1 - [F8WEL7_HUMAN] | 1,32 |
| Q13542 | Eukaryotic translation initiation factor 4E-binding protein 2 OS=Homo sapiens GN=EIF4EBP2 PE=1 SV=1 - [4EBP2_HUMAN] | 1,32 |
| P61081 | NEDD8-conjugating enzyme Ubc12 OS=Homo sapiens GN=UBE2M PE=1 SV=1 - [UBC12_HUMAN] | 1,31 |
| M0R3D4 | Prenylated Rab acceptor protein 1 OS=Homo sapiens GN=RABAC1 PE=1 SV=1 - [M0R3D4_HUMAN] | 1,31 |
| P51812 | Ribosomal protein S6 kinase alpha-3 OS=Homo sapiens GN=RPS6KA3 PE=1 SV=1 - [KS6A3_HUMAN] | 1,31 |
| H7C2A6 | Cysteine protease ATG4B (Fragment) OS=Homo sapiens GN=ATG4B PE=1 SV=1 - [H7C2A6_HUMAN] | 1,30 |
| P62495-2 | Isoform 2 of Eukaryotic peptide chain release factor subunit 1 OS=Homo sapiens GN=ETF1 - [ERF1_HUMAN] | 1,30 |
| P82673 | 28S ribosomal protein S35, mitochondrial OS=Homo sapiens GN=MRPS35 PE=1 SV=1 - [RT35_HUMAN] | 1,30 |
| O60603 | Toll-like receptor 2 OS=Homo sapiens GN=TLR2 PE=1 SV=1 - [TLR2_HUMAN] | 1,30 |

| **D) Separated Proteomics setup: Soluble fraction**  **Potential miR-328 targets** | | |
| --- | --- | --- |
| **Accession** | **Description** | **TMT ratio** |
| M0QXH0 | Thioredoxin, mitochondrial OS=Homo sapiens GN=TXN2 PE=1 SV=1 - [M0QXH0_HUMAN] | 1,57 |
| Q7Z7H8 | 39S ribosomal protein L10, mitochondrial OS=Homo sapiens GN=MRPL10 PE=1 SV=3 - [RM10_HUMAN] | 1,55 |
| H0Y2X1 | Chromosome 17 open reading frame 62, isoform CRA_c OS=Homo sapiens GN=C17orf62 PE=1 SV=1 - [H0Y2X1_HUMAN] | 1,52 |
| P07204 | Thrombomodulin OS=Homo sapiens GN=THBD PE=1 SV=2 - [TRBM_HUMAN] | 1,48 |
| F5GYT3 | Lysophospholipid acyltransferase 5 OS=Homo sapiens GN=LPCAT3 PE=1 SV=1 - [F5GYT3_HUMAN] | 1,46 |
| P46977 | Dolichyl-diphosphooligosaccharide--protein glycosyltransferase subunit STT3A OS=Homo sapiens GN=STT3A PE=1 SV=2 - [STT3A_HUMAN] | 1,38 |
| P55854 | Small ubiquitin-related modifier 3 OS=Homo sapiens GN=SUMO3 PE=1 SV=2 - [SUMO3_HUMAN] | 1,35 |
| P07602 | Prosaposin OS=Homo sapiens GN=PSAP PE=1 SV=2 - [SAP_HUMAN] | 1,34 |
| Q9NUQ2 | 1-acyl-sn-glycerol-3-phosphate acyltransferase epsilon OS=Homo sapiens GN=AGPAT5 PE=1 SV=3 - [PLCE_HUMAN] | 1,33 |
| B4E3S0 | Coronin OS=Homo sapiens GN=CORO1C PE=1 SV=1 - [B4E3S0_HUMAN] | 1,30 |
| Q14849-2 | Isoform 2 of StAR-related lipid transfer protein 3 OS=Homo sapiens GN=STARD3 - [STAR3_HUMAN] | 1,30 |
| A0A075B6F6 | Minor histocompatibility antigen H13 (Fragment) OS=Homo sapiens GN=HM13 PE=1 SV=1 - [A0A075B6F6_HUMAN] | 1,30 |
| Q969M1 | Mitochondrial import receptor subunit TOM40B OS=Homo sapiens GN=TOMM40L PE=2 SV=1 - [TM40L_HUMAN] | 1,30 |
| K7EK07 | Histone H3 (Fragment) OS=Homo sapiens GN=H3F3B PE=1 SV=1 - [K7EK07_HUMAN] | 1,30 |
| F5GX39 | Transmembrane emp24 domain-containing protein 2 OS=Homo sapiens GN=TMED2 PE=1 SV=1 - [F5GX39_HUMAN] | 1,30 |

**Table S2. Potential hnRNP E2/miR-328 targets detected in TMT-based proteomics study with pooled setup** (A-B). A) soluble fraction and B) microsomal fraction. Potential hnRNP E2/miR-328 targets detected in TMT-based proteomics study with separated setup (C-D).

| **A) Pooled Proteomics setup: Soluble fraction**  **Potential hnRNP E2/miR-328 targets** | | |  |
| --- | --- | --- | --- |
| **Accession** | **Description** | **TMT ratio** | **5´UTR intron** |
| P14854 | Cytochrome c oxidase subunit 6B1 OS=Homo sapiens GN=COX6B1 PE=1 SV=2 - [CX6B1_HUMAN] | 0,77 | yes |
| Q9NPB8 | Glycerophosphocholine phosphodiesterase GPCPD1 OS=Homo sapiens GN=GPCPD1 PE=1 SV=2 - [GPCP1_HUMAN] | 0,77 | yes |
| P82970 | High mobility group nucleosome-binding domain-containing protein 5 OS=Homo sapiens GN=HMGN5 PE=1 SV=1 - [HMGN5_HUMAN] | 0,77 | yes |
| Q9NTZ6 | RNA-binding protein 12 OS=Homo sapiens GN=RBM12 PE=1 SV=1 - [RBM12_HUMAN] | 0,77 | yes |
| Q9H6U6-6 | Isoform 6 of Breast carcinoma-amplified sequence 3 OS=Homo sapiens GN=BCAS3 - [BCAS3_HUMAN] | 0,77 | yes |
| P43405-2 | Isoform Short of Tyrosine-protein kinase SYK OS=Homo sapiens GN=SYK - [KSYK_HUMAN] | 0,76 | yes |
| Q9P1Y6-2 | Isoform 2 of PHD and RING finger domain-containing protein 1 OS=Homo sapiens GN=PHRF1 - [PHRF1_HUMAN] | 0,76 | yes |
| Q02040 | A-kinase anchor protein 17A OS=Homo sapiens GN=AKAP17A PE=1 SV=2 - [AK17A_HUMAN] | 0,76 | yes |
| P16885 | 1-phosphatidylinositol 4,5-bisphosphate phosphodiesterase gamma-2 OS=Homo sapiens GN=PLCG2 PE=1 SV=4 - [PLCG2_HUMAN] | 0,75 | yes |
| Q9P0U3-2 | Isoform 2 of Sentrin-specific protease 1 OS=Homo sapiens GN=SENP1 - [SENP1_HUMAN] | 0,75 | yes |
| Q9NVN8 | Guanine nucleotide-binding protein-like 3-like protein OS=Homo sapiens GN=GNL3L PE=1 SV=1 - [GNL3L_HUMAN] | 0,75 | yes |
| P51956-2 | Isoform 2 of Serine/threonine-protein kinase Nek3 OS=Homo sapiens GN=NEK3 - [NEK3_HUMAN] | 0,75 | yes |
| P08621-2 | Isoform 2 of U1 small nuclear ribonucleoprotein 70 kDa OS=Homo sapiens GN=SNRNP70 - [RU17_HUMAN] | 0,74 | yes |
| Q7L8L6 | FAST kinase domain-containing protein 5 OS=Homo sapiens GN=FASTKD5 PE=1 SV=1 - [FAKD5_HUMAN] | 0,74 | yes |
| P29692 | Elongation factor 1-delta OS=Homo sapiens GN=EEF1D PE=1 SV=5 - [EF1D_HUMAN] | 0,74 | yes |
| P22314-2 | Isoform 2 of Ubiquitin-like modifier-activating enzyme 1 OS=Homo sapiens GN=UBA1 - [UBA1_HUMAN] | 0,73 | yes |
| P78330 | Phosphoserine phosphatase OS=Homo sapiens GN=PSPH PE=1 SV=2 - [SERB_HUMAN] | 0,73 | yes |
| P50583 | Bis(5'-nucleosyl)-tetraphosphatase [asymmetrical] OS=Homo sapiens GN=NUDT2 PE=1 SV=3 - [AP4A_HUMAN] | 0,73 | yes |
| P42680 | Tyrosine-protein kinase Tec OS=Homo sapiens GN=TEC PE=1 SV=2 - [TEC_HUMAN] | 0,72 | yes |
| Q9Y3L5 | Ras-related protein Rap-2c OS=Homo sapiens GN=RAP2C PE=1 SV=1 - [RAP2C_HUMAN] | 0,71 | yes |
| Q96Q11-2 | Isoform 2 of CCA tRNA nucleotidyltransferase 1, mitochondrial OS=Homo sapiens GN=TRNT1 - [TRNT1_HUMAN] | 0,71 | yes |
| Q06587-2 | Isoform 2 of E3 ubiquitin-protein ligase RING1 OS=Homo sapiens GN=RING1 - [RING1_HUMAN] | 0,71 | yes |
| Q9BU89 | Deoxyhypusine hydroxylase OS=Homo sapiens GN=DOHH PE=1 SV=1 - [DOHH_HUMAN] | 0,70 | yes |
| P04083 | Annexin A1 OS=Homo sapiens GN=ANXA1 PE=1 SV=2 - [ANXA1_HUMAN] | 0,70 | yes |
| P09525 | Annexin A4 OS=Homo sapiens GN=ANXA4 PE=1 SV=4 - [ANXA4_HUMAN] | 0,69 | yes |
| P63167 | Dynein light chain 1, cytoplasmic OS=Homo sapiens GN=DYNLL1 PE=1 SV=1 - [DYL1_HUMAN] | 0,69 | yes |
| P55795 | Heterogeneous nuclear ribonucleoprotein H2 OS=Homo sapiens GN=HNRNPH2 PE=1 SV=1 - [HNRH2_HUMAN] | 0,69 | yes |
| Q6T4P5-4 | Isoform 4 of Lipid phosphate phosphatase-related protein type 3 OS=Homo sapiens GN=LPPR3 - [LPPR3_HUMAN] | 0,68 | yes |
| Q8WVY7 | Ubiquitin-like domain-containing CTD phosphatase 1 OS=Homo sapiens GN=UBLCP1 PE=1 SV=2 - [UBCP1_HUMAN] | 0,66 | yes |
| Q969M7-5 | Isoform 5 of NEDD8-conjugating enzyme UBE2F OS=Homo sapiens GN=UBE2F - [UBE2F_HUMAN] | 0,66 | yes |
| Q9BRX2 | Protein pelota homolog OS=Homo sapiens GN=PELO PE=1 SV=2 - [PELO_HUMAN] | 0,65 | yes |
| P00966 | Argininosuccinate synthase OS=Homo sapiens GN=ASS1 PE=1 SV=2 - [ASSY_HUMAN] | 0,64 | yes |
| Q15428 | Splicing factor 3A subunit 2 OS=Homo sapiens GN=SF3A2 PE=1 SV=2 - [SF3A2_HUMAN] | 0,61 | yes |
| P27695 | DNA-(apurinic or apyrimidinic site) lyase OS=Homo sapiens GN=APEX1 PE=1 SV=2 - [APEX1_HUMAN] | 0,58 | yes |
| P29692-3 | Isoform 3 of Elongation factor 1-delta OS=Homo sapiens GN=EEF1D - [EF1D_HUMAN] | 0,58 | yes |
| O95785-2 | Isoform 2 of Protein Wiz OS=Homo sapiens GN=WIZ - [WIZ_HUMAN] | 0,57 | yes |
| P18754 | Regulator of chromosome condensation OS=Homo sapiens GN=RCC1 PE=1 SV=1 - [RCC1_HUMAN] | 0,57 | yes |
| P14324-2 | Isoform 2 of Farnesyl pyrophosphate synthase OS=Homo sapiens GN=FDPS - [FPPS_HUMAN] | 0,48 | yes |
| P35813 | Protein phosphatase 1A OS=Homo sapiens GN=PPM1A PE=1 SV=1 - [PPM1A_HUMAN] | 0,73 | yes |
| Q9H9Y6 | DNA-directed RNA polymerase I subunit RPA2 OS=Homo sapiens GN=POLR1B PE=1 SV=2 - [RPA2_HUMAN] | 0,77 | no |
| Q5TBB1-2 | Isoform 2 of Ribonuclease H2 subunit B OS=Homo sapiens GN=RNASEH2B - [RNH2B_HUMAN] | 0,77 | no |
| P57076 | UPF0769 protein C21orf59 OS=Homo sapiens GN=C21orf59 PE=1 SV=1 - [CU059_HUMAN] | 0,77 | no |
| P13995-2 | Isoform 2 of Bifunctional methylenetetrahydrofolate dehydrogenase/cyclohydrolase, mitochondrial OS=Homo sapiens GN=MTHFD2 - [MTDC_HUMAN] | 0,76 | no |
| Q9C000-6 | Isoform 6 of NACHT, LRR and PYD domains-containing protein 1 OS=Homo sapiens GN=NLRP1 - [NALP1_HUMAN] | 0,76 | no |
| P42785 | Lysosomal Pro-X carboxypeptidase OS=Homo sapiens GN=PRCP PE=1 SV=1 - [PCP_HUMAN] | 0,76 | no |
| Q96C86 | m7GpppX diphosphatase OS=Homo sapiens GN=DCPS PE=1 SV=2 - [DCPS_HUMAN] | 0,76 | no |
| P62253 | Ubiquitin-conjugating enzyme E2 G1 OS=Homo sapiens GN=UBE2G1 PE=1 SV=3 - [UB2G1_HUMAN] | 0,75 | no |
| P49411 | Elongation factor Tu, mitochondrial OS=Homo sapiens GN=TUFM PE=1 SV=2 - [EFTU_HUMAN] | 0,75 | no |
| Q13151 | Heterogeneous nuclear ribonucleoprotein A0 OS=Homo sapiens GN=HNRNPA0 PE=1 SV=1 - [ROA0_HUMAN] | 0,74 | no |
| O75400-2 | Isoform 2 of Pre-mRNA-processing factor 40 homolog A OS=Homo sapiens GN=PRPF40A - [PR40A_HUMAN] | 0,74 | no |
| O95989 | Diphosphoinositol polyphosphate phosphohydrolase 1 OS=Homo sapiens GN=NUDT3 PE=1 SV=1 - [NUDT3_HUMAN] | 0,73 | no |
| Q86V21 | Acetoacetyl-CoA synthetase OS=Homo sapiens GN=AACS PE=1 SV=1 - [AACS_HUMAN] | 0,72 | no |
| Q9UHY7 | Enolase-phosphatase E1 OS=Homo sapiens GN=ENOPH1 PE=1 SV=1 - [ENOPH_HUMAN] | 0,71 | no |
| Q8IVM0 | Coiled-coil domain-containing protein 50 OS=Homo sapiens GN=CCDC50 PE=1 SV=1 - [CCD50_HUMAN] | 0,70 | no |
| P10619-2 | Isoform 2 of Lysosomal protective protein OS=Homo sapiens GN=CTSA - [PPGB_HUMAN] | 0,69 | no |
| P21266 | Glutathione S-transferase Mu 3 OS=Homo sapiens GN=GSTM3 PE=1 SV=3 - [GSTM3_HUMAN] | 0,68 | no |
| Q8N5U6 | RING finger protein 10 OS=Homo sapiens GN=RNF10 PE=1 SV=2 - [RNF10_HUMAN] | 0,68 | no |
| Q16584 | Mitogen-activated protein kinase kinase kinase 11 OS=Homo sapiens GN=MAP3K11 PE=1 SV=1 - [M3K11_HUMAN] | 0,66 | no |
| Q9H3M0 | Potassium voltage-gated channel subfamily F member 1 OS=Homo sapiens GN=KCNF1 PE=1 SV=1 - [KCNF1_HUMAN] | 0,66 | no |
| O75390 | Citrate synthase, mitochondrial OS=Homo sapiens GN=CS PE=1 SV=2 - [CISY_HUMAN] | 0,66 | no |
| Q86WC6 | Protein phosphatase 1 regulatory subunit 27 OS=Homo sapiens GN=PPP1R27 PE=1 SV=1 - [PPR27_HUMAN] | 0,65 | no |
| Q969G6 | Riboflavin kinase OS=Homo sapiens GN=RFK PE=1 SV=2 - [RIFK_HUMAN] | 0,64 | no |
| Q9Y3C6 | Peptidyl-prolyl cis-trans isomerase-like 1 OS=Homo sapiens GN=PPIL1 PE=1 SV=1 - [PPIL1_HUMAN] | 0,62 | no |
| Q92636 | Protein FAN OS=Homo sapiens GN=NSMAF PE=1 SV=2 - [FAN_HUMAN] | 0,57 | no |
| P40425 | Pre-B-cell leukemia transcription factor 2 OS=Homo sapiens GN=PBX2 PE=1 SV=2 - [PBX2_HUMAN] | 0,44 | no |
| Q8WTS6 | Histone-lysine N-methyltransferase SETD7 OS=Homo sapiens GN=SETD7 PE=1 SV=1 - [SETD7_HUMAN] | 0,16 | no |

| **B) Pooled Proteomics setup: Microsomal fraction**  **Potential hnRNP E2/miR-328 targets** | | |  |
| --- | --- | --- | --- |
| **Accession** | **Description** | **TMT ratio** | **5´UTR intron** |
| P48634-2 | Isoform 2 of Protein PRRC2A OS=Homo sapiens GN=PRRC2A - [PRC2A_HUMAN] | 0,77 | yes |
| P78332 | RNA-binding protein 6 OS=Homo sapiens GN=RBM6 PE=1 SV=5 - [RBM6_HUMAN] | 0,77 | yes |
| Q5T6F2-2 | Isoform 2 of Ubiquitin-associated protein 2 OS=Homo sapiens GN=UBAP2 - [UBAP2_HUMAN] | 0,77 | yes |
| Q9BUT1 | 3-hydroxybutyrate dehydrogenase type 2 OS=Homo sapiens GN=BDH2 PE=1 SV=2 - [BDH2_HUMAN] | 0,77 | yes |
| Q86UP2-4 | Isoform 4 of Kinectin OS=Homo sapiens GN=KTN1 - [KTN1_HUMAN] | 0,77 | yes |
| O75531 | Barrier-to-autointegration factor OS=Homo sapiens GN=BANF1 PE=1 SV=1 - [BAF_HUMAN] | 0,77 | yes |
| Q16831 | Uridine phosphorylase 1 OS=Homo sapiens GN=UPP1 PE=1 SV=1 - [UPP1_HUMAN] | 0,77 | yes |
| Q14157 | Ubiquitin-associated protein 2-like OS=Homo sapiens GN=UBAP2L PE=1 SV=2 - [UBP2L_HUMAN] | 0,77 | yes |
| Q8N300 | Small vasohibin-binding protein OS=Homo sapiens GN=SVBP PE=1 SV=1 - [SVBP_HUMAN] | 0,77 | yes |
| Q9BTL3 | RNMT-activating mini protein OS=Homo sapiens GN=FAM103A1 PE=1 SV=1 - [RAM_HUMAN] | 0,77 | yes |
| P07108 | Acyl-CoA-binding protein OS=Homo sapiens GN=DBI PE=1 SV=2 - [ACBP_HUMAN] | 0,77 | yes |
| Q96BW5-2 | Isoform 2 of Phosphotriesterase-related protein OS=Homo sapiens GN=PTER - [PTER_HUMAN] | 0,77 | yes |
| Q96FJ2 | Dynein light chain 2, cytoplasmic OS=Homo sapiens GN=DYNLL2 PE=1 SV=1 - [DYL2_HUMAN] | 0,76 | yes |
| Q9P1F3 | Costars family protein ABRACL OS=Homo sapiens GN=ABRACL PE=1 SV=1 - [ABRAL_HUMAN] | 0,76 | yes |
| Q8IZ21-3 | Isoform 3 of Phosphatase and actin regulator 4 OS=Homo sapiens GN=PHACTR4 - [PHAR4_HUMAN] | 0,75 | yes |
| P06702 | Protein S100-A9 OS=Homo sapiens GN=S100A9 PE=1 SV=1 - [S10A9_HUMAN] | 0,74 | yes |
| P09429 | High mobility group protein B1 OS=Homo sapiens GN=HMGB1 PE=1 SV=3 - [HMGB1_HUMAN] | 0,74 | yes |
| O95696 | Bromodomain-containing protein 1 OS=Homo sapiens GN=BRD1 PE=1 SV=1 - [BRD1_HUMAN] | 0,74 | yes |
| P26447 | Protein S100-A4 OS=Homo sapiens GN=S100A4 PE=1 SV=1 - [S10A4_HUMAN] | 0,73 | yes |
| Q8IYB7-4 | Isoform 4 of DIS3-like exonuclease 2 OS=Homo sapiens GN=DIS3L2 - [DI3L2_HUMAN] | 0,73 | yes |
| P39210 | Protein Mpv17 OS=Homo sapiens GN=MPV17 PE=1 SV=1 - [MPV17_HUMAN] | 0,73 | yes |
| P18859 | ATP synthase-coupling factor 6, mitochondrial OS=Homo sapiens GN=ATP5J PE=1 SV=1 - [ATP5J_HUMAN] | 0,72 | yes |
| Q86UP2-2 | Isoform 2 of Kinectin OS=Homo sapiens GN=KTN1 - [KTN1_HUMAN] | 0,71 | yes |
| P50238 | Cysteine-rich protein 1 OS=Homo sapiens GN=CRIP1 PE=1 SV=3 - [CRIP1_HUMAN] | 0,71 | yes |
| Q2TAC2 | Coiled-coil domain-containing protein 57 OS=Homo sapiens GN=CCDC57 PE=1 SV=2 - [CCD57_HUMAN] | 0,71 | yes |
| Q9C0C2 | 182 kDa tankyrase-1-binding protein OS=Homo sapiens GN=TNKS1BP1 PE=1 SV=4 - [TB182_HUMAN] | 0,71 | yes |
| Q7L266-2 | Isoform 2 of Isoaspartyl peptidase/L-asparaginase OS=Homo sapiens GN=ASRGL1 - [ASGL1_HUMAN] | 0,70 | yes |
| P49789 | Bis(5'-adenosyl)-triphosphatase OS=Homo sapiens GN=FHIT PE=1 SV=3 - [FHIT_HUMAN] | 0,70 | yes |
| Q53EZ4-2 | Isoform 2 of Centrosomal protein of 55 kDa OS=Homo sapiens GN=CEP55 - [CEP55_HUMAN] | 0,70 | yes |
| Q9BV29 | Uncharacterized protein C15orf57 OS=Homo sapiens GN=C15orf57 PE=1 SV=2 - [CO057_HUMAN] | 0,70 | yes |
| Q6FIF0-2 | Isoform 2 of AN1-type zinc finger protein 6 OS=Homo sapiens GN=ZFAND6 - [ZFAN6_HUMAN] | 0,70 | yes |
| Q96RL1-3 | Isoform 3 of BRCA1-A complex subunit RAP80 OS=Homo sapiens GN=UIMC1 - [UIMC1_HUMAN] | 0,69 | yes |
| Q9BS18 | Anaphase-promoting complex subunit 13 OS=Homo sapiens GN=ANAPC13 PE=1 SV=1 - [APC13_HUMAN] | 0,67 | yes |
| Q562F6-2 | Isoform 2 of Shugoshin-like 2 OS=Homo sapiens GN=SGOL2 - [SGOL2_HUMAN] | 0,67 | yes |
| P02656 | Apolipoprotein C-III OS=Homo sapiens GN=APOC3 PE=1 SV=1 - [APOC3_HUMAN] | 0,66 | yes |
| Q9P275 | Ubiquitin carboxyl-terminal hydrolase 36 OS=Homo sapiens GN=USP36 PE=1 SV=3 - [UBP36_HUMAN] | 0,66 | yes |
| P14854 | Cytochrome c oxidase subunit 6B1 OS=Homo sapiens GN=COX6B1 PE=1 SV=2 - [CX6B1_HUMAN] | 0,66 | yes |
| Q96LB3-2 | Isoform 2 of Intraflagellar transport protein 74 homolog OS=Homo sapiens GN=IFT74 - [IFT74_HUMAN] | 0,66 | yes |
| P56378 | 6.8 kDa mitochondrial proteolipid OS=Homo sapiens GN=MP68 PE=1 SV=1 - [68MP_HUMAN] | 0,63 | yes |
| Q9NRP2 | COX assembly mitochondrial protein 2 homolog OS=Homo sapiens GN=CMC2 PE=1 SV=1 - [COXM2_HUMAN] | 0,61 | yes |
| Q96SI9-2 | Isoform 2 of Spermatid perinuclear RNA-binding protein OS=Homo sapiens GN=STRBP - [STRBP_HUMAN] | 0,61 | yes |
| O95835-2 | Isoform 2 of Serine/threonine-protein kinase LATS1 OS=Homo sapiens GN=LATS1 - [LATS1_HUMAN] | 0,53 | yes |
| Q6NXR4 | TELO2-interacting protein 2 OS=Homo sapiens GN=TTI2 PE=1 SV=1 - [TTI2_HUMAN] | 0,66 | yes |
| P42785 | Lysosomal Pro-X carboxypeptidase OS=Homo sapiens GN=PRCP PE=1 SV=1 - [PCP_HUMAN] | 0,77 | no |
| O43768-6 | Isoform 6 of Alpha-endosulfine OS=Homo sapiens GN=ENSA - [ENSA_HUMAN] | 0,77 | no |
| Q6JBY9 | CapZ-interacting protein OS=Homo sapiens GN=RCSD1 PE=1 SV=1 - [CPZIP_HUMAN] | 0,77 | no |
| Q9H6Y2-2 | Isoform 2 of WD repeat-containing protein 55 OS=Homo sapiens GN=WDR55 - [WDR55_HUMAN] | 0,77 | no |
| Q9NYV4-3 | Isoform 3 of Cyclin-dependent kinase 12 OS=Homo sapiens GN=CDK12 - [CDK12_HUMAN] | 0,77 | no |
| P02774-2 | Isoform 2 of Vitamin D-binding protein OS=Homo sapiens GN=GC - [VTDB_HUMAN] | 0,77 | no |
| Q96F46-2 | Isoform 2 of Interleukin-17 receptor A OS=Homo sapiens GN=IL17RA - [I17RA_HUMAN] | 0,77 | no |
| O75410-7 | Isoform 7 of Transforming acidic coiled-coil-containing protein 1 OS=Homo sapiens GN=TACC1 - [TACC1_HUMAN] | 0,76 | no |
| Q8NBM4-4 | Isoform 4 of Ubiquitin-associated domain-containing protein 2 OS=Homo sapiens GN=UBAC2 - [UBAC2_HUMAN] | 0,76 | no |
| P23434 | Glycine cleavage system H protein, mitochondrial OS=Homo sapiens GN=GCSH PE=1 SV=2 - [GCSH_HUMAN] | 0,76 | no |
| Q9NWT1 | p21-activated protein kinase-interacting protein 1 OS=Homo sapiens GN=PAK1IP1 PE=1 SV=2 - [PK1IP_HUMAN] | 0,76 | no |
| Q9Y3C1 | Nucleolar protein 16 OS=Homo sapiens GN=NOP16 PE=1 SV=2 - [NOP16_HUMAN] | 0,76 | no |
| P01137 | Transforming growth factor beta-1 OS=Homo sapiens GN=TGFB1 PE=1 SV=2 - [TGFB1_HUMAN] | 0,75 | no |
| P36954 | DNA-directed RNA polymerase II subunit RPB9 OS=Homo sapiens GN=POLR2I PE=1 SV=1 - [RPB9_HUMAN] | 0,75 | no |
| P61604 | 10 kDa heat shock protein, mitochondrial OS=Homo sapiens GN=HSPE1 PE=1 SV=2 - [CH10_HUMAN] | 0,75 | no |
| Q8NDH3-5 | Isoform 5 of Probable aminopeptidase NPEPL1 OS=Homo sapiens GN=NPEPL1 - [PEPL1_HUMAN] | 0,75 | no |
| Q5T3J3 | Ligand-dependent nuclear receptor-interacting factor 1 OS=Homo sapiens GN=LRIF1 PE=1 SV=1 - [LRIF1_HUMAN] | 0,75 | no |
| P04004 | Vitronectin OS=Homo sapiens GN=VTN PE=1 SV=1 - [VTNC_HUMAN] | 0,75 | no |
| Q02487-2 | Isoform 2B of Desmocollin-2 OS=Homo sapiens GN=DSC2 - [DSC2_HUMAN] | 0,74 | no |
| Q99848 | Probable rRNA-processing protein EBP2 OS=Homo sapiens GN=EBNA1BP2 PE=1 SV=2 - [EBP2_HUMAN] | 0,73 | no |
| O95429-2 | Isoform 2 of BAG family molecular chaperone regulator 4 OS=Homo sapiens GN=BAG4 - [BAG4_HUMAN] | 0,73 | no |
| O43678 | NADH dehydrogenase [ubiquinone] 1 alpha subcomplex subunit 2 OS=Homo sapiens GN=NDUFA2 PE=1 SV=3 - [NDUA2_HUMAN] | 0,73 | no |
| Q8N5U6 | RING finger protein 10 OS=Homo sapiens GN=RNF10 PE=1 SV=2 - [RNF10_HUMAN] | 0,71 | no |
| P02794 | Ferritin heavy chain OS=Homo sapiens GN=FTH1 PE=1 SV=2 - [FRIH_HUMAN] | 0,70 | no |
| Q04721 | Neurogenic locus notch homolog protein 2 OS=Homo sapiens GN=NOTCH2 PE=1 SV=3 - [NOTC2_HUMAN] | 0,70 | no |
| Q96MU7-2 | Isoform 2 of YTH domain-containing protein 1 OS=Homo sapiens GN=YTHDC1 - [YTDC1_HUMAN] | 0,70 | no |
| O60927 | Protein phosphatase 1 regulatory subunit 11 OS=Homo sapiens GN=PPP1R11 PE=1 SV=1 - [PP1RB_HUMAN] | 0,70 | no |
| P27540-2 | Isoform 2 of Aryl hydrocarbon receptor nuclear translocator OS=Homo sapiens GN=ARNT - [ARNT_HUMAN] | 0,69 | no |
| O75179-4 | Isoform 4 of Ankyrin repeat domain-containing protein 17 OS=Homo sapiens GN=ANKRD17 - [ANR17_HUMAN] | 0,69 | no |
| Q9NUL7 | Probable ATP-dependent RNA helicase DDX28 OS=Homo sapiens GN=DDX28 PE=1 SV=2 - [DDX28_HUMAN] | 0,62 | no |
| P60896 | 26S proteasome complex subunit DSS1 OS=Homo sapiens GN=SHFM1 PE=1 SV=1 - [DSS1_HUMAN] | 0,60 | no |
| P11217-2 | Isoform 2 of Glycogen phosphorylase, muscle form OS=Homo sapiens GN=PYGM - [PYGM_HUMAN] | 0,43 | no |

| **C) Separated Proteomics setup: Soluble fraction**  **Potential hnRNP E2/miR-328 targets** | | |  |
| --- | --- | --- | --- |
| **Accession** | **Description** | **TMT ratio** | **5´UTR intron** |
| Q99674 | Cell growth regulator with EF hand domain protein 1 OS=Homo sapiens GN=CGREF1 PE=2 SV=2 - [CGRE1_HUMAN] | 0,76 | yes |
| E5RK24 | Transcription initiation factor IIE subunit beta OS=Homo sapiens GN=GTF2E2 PE=1 SV=1 - [E5RK24_HUMAN] | 0,74 | yes |
| B4DJ81 | NADH-ubiquinone oxidoreductase 75 kDa subunit, mitochondrial OS=Homo sapiens GN=NDUFS1 PE=1 SV=1 - [B4DJ81_HUMAN] | 0,72 | yes |
| D6R9M3 | BRCA1-A complex subunit RAP80 (Fragment) OS=Homo sapiens GN=UIMC1 PE=1 SV=1 - [D6R9M3_HUMAN] | 0,70 | yes |
| B9EGQ5 | Protein Wiz OS=Homo sapiens GN=WIZ PE=1 SV=1 - [B9EGQ5_HUMAN] | 0,69 | yes |
| K7EMV3 | Histone H3 OS=Homo sapiens GN=H3F3B PE=1 SV=1 - [K7EMV3_HUMAN] | 0,63 | yes |
| F5H5A1 | Acyl-CoA synthetase family member 3, mitochondrial OS=Homo sapiens GN=ACSF3 PE=1 SV=1 - [F5H5A1_HUMAN] | 0,57 | yes |
| F8WAJ0 | Probable ATP-dependent RNA helicase DDX31 OS=Homo sapiens GN=DDX31 PE=1 SV=2 - [F8WAJ0_HUMAN] | 0,74 | no |
| P0CG38 | POTE ankyrin domain family member I OS=Homo sapiens GN=POTEI PE=3 SV=1 - [POTEI_HUMAN] | 0,73 | no |
| O43678 | NADH dehydrogenase [ubiquinone] 1 alpha subcomplex subunit 2 OS=Homo sapiens GN=NDUFA2 PE=1 SV=3 - [NDUA2_HUMAN] | 0,68 | no |
| P68371 | Tubulin beta-4B chain OS=Homo sapiens GN=TUBB4B PE=1 SV=1 - [TBB4B_HUMAN] | 0,68 | no |
| Q8IU81 | Interferon regulatory factor 2-binding protein 1 OS=Homo sapiens GN=IRF2BP1 PE=1 SV=1 - [I2BP1_HUMAN] | 0,47 | no |

| **D) Separated Proteomics setup: Microsomal fraction**  **Potential hnRNP E2/miR-328 targets** | | |  |
| --- | --- | --- | --- |
| **Accession** | **Description** | **TMT ratio** | **5´UTR intron** |
| Q9UNS1-2 | Isoform 2 of Protein timeless homolog OS=Homo sapiens GN=TIMELESS - [TIM_HUMAN] | 0,77 | yes |
| Q03154-2 | Isoform 2 of Aminoacylase-1 OS=Homo sapiens GN=ACY1 - [ACY1_HUMAN] | 0,77 | yes |
| E9PK09 | Bcl-2-associated transcription factor 1 (Fragment) OS=Homo sapiens GN=BCLAF1 PE=1 SV=5 - [E9PK09_HUMAN] | 0,77 | yes |
| Q01518 | Adenylyl cyclase-associated protein 1 OS=Homo sapiens GN=CAP1 PE=1 SV=5 - [CAP1_HUMAN] | 0,76 | yes |
| G3V5Q1 | DNA-(apurinic or apyrimidinic site) lyase (Fragment) OS=Homo sapiens GN=APEX1 PE=1 SV=1 - [G3V5Q1_HUMAN] | 0,76 | yes |
| Q9UNF0-2 | Isoform 2 of Protein kinase C and casein kinase substrate in neurons protein 2 OS=Homo sapiens GN=PACSIN2 - [PACN2_HUMAN] | 0,75 | yes |
| C9JAB9 | Cytoplasmic protein NCK1 (Fragment) OS=Homo sapiens GN=NCK1 PE=1 SV=1 - [C9JAB9_HUMAN] | 0,75 | yes |
| Q02218-2 | Isoform 2 of 2-oxoglutarate dehydrogenase, mitochondrial OS=Homo sapiens GN=OGDH - [ODO1_HUMAN] | 0,74 | yes |
| Q5JUR7-2 | Isoform 2 of Testis-expressed sequence 30 protein OS=Homo sapiens GN=TEX30 - [TEX30_HUMAN] | 0,74 | yes |
| P43405-2 | Isoform Short of Tyrosine-protein kinase SYK OS=Homo sapiens GN=SYK - [KSYK_HUMAN] | 0,74 | yes |
| P14550 | Alcohol dehydrogenase [NADP(+)] OS=Homo sapiens GN=AKR1A1 PE=1 SV=3 - [AK1A1_HUMAN] | 0,74 | yes |
| P07858 | Cathepsin B OS=Homo sapiens GN=CTSB PE=1 SV=3 - [CATB_HUMAN] | 0,74 | yes |
| C9JRS7 | CCA tRNA nucleotidyltransferase 1, mitochondrial (Fragment) OS=Homo sapiens GN=TRNT1 PE=1 SV=1 - [C9JRS7_HUMAN] | 0,74 | yes |
| P19474-2 | Isoform 2 of E3 ubiquitin-protein ligase TRIM21 OS=Homo sapiens GN=TRIM21 - [RO52_HUMAN] | 0,73 | yes |
| Q9NPB8 | Glycerophosphocholine phosphodiesterase GPCPD1 OS=Homo sapiens GN=GPCPD1 PE=1 SV=2 - [GPCP1_HUMAN] | 0,73 | yes |
| P10155-2 | Isoform Short of 60 kDa SS-A/Ro ribonucleoprotein OS=Homo sapiens GN=TROVE2 - [RO60_HUMAN] | 0,73 | yes |
| Q5T6L5 | Argininosuccinate synthase (Fragment) OS=Homo sapiens GN=ASS1 PE=1 SV=1 - [Q5T6L5_HUMAN] | 0,71 | yes |
| P09525 | Annexin A4 OS=Homo sapiens GN=ANXA4 PE=1 SV=4 - [ANXA4_HUMAN] | 0,70 | yes |
| Q6UWE0-3 | Isoform 3 of E3 ubiquitin-protein ligase LRSAM1 OS=Homo sapiens GN=LRSAM1 - [LRSM1_HUMAN] | 0,68 | yes |
| P35813-2 | Isoform Alpha-2 of Protein phosphatase 1A OS=Homo sapiens GN=PPM1A - [PPM1A_HUMAN] | 0,65 | yes |
| M0QYR1 | U1 small nuclear ribonucleoprotein 70 kDa (Fragment) OS=Homo sapiens GN=SNRNP70 PE=1 SV=1 - [M0QYR1_HUMAN] | 0,64 | yes |
| P21266 | Glutathione S-transferase Mu 3 OS=Homo sapiens GN=GSTM3 PE=1 SV=3 - [GSTM3_HUMAN] | 0,63 | yes |
| P39019 | 40S ribosomal protein S19 OS=Homo sapiens GN=RPS19 PE=1 SV=2 - [RS19_HUMAN] | 0,63 | yes |
| P22314-2 | Isoform 2 of Ubiquitin-like modifier-activating enzyme 1 OS=Homo sapiens GN=UBA1 - [UBA1_HUMAN] | 0,62 | yes |
| E9PR95 | Ester hydrolase C11orf54 (Fragment) OS=Homo sapiens GN=C11orf54 PE=1 SV=5 - [E9PR95_HUMAN] | 0,60 | yes |
| P04083 | Annexin A1 OS=Homo sapiens GN=ANXA1 PE=1 SV=2 - [ANXA1_HUMAN] | 0,56 | yes |
| P14324-2 | Isoform 2 of Farnesyl pyrophosphate synthase OS=Homo sapiens GN=FDPS - [FPPS_HUMAN] | 0,44 | yes |
| P52565-2 | Isoform 2 of Rho GDP-dissociation inhibitor 1 OS=Homo sapiens GN=ARHGDIA - [GDIR1_HUMAN] | 0,44 | yes |
| Q9BYD6 | 39S ribosomal protein L1, mitochondrial OS=Homo sapiens GN=MRPL1 PE=1 SV=2 - [RM01_HUMAN] | 0,77 | no |
| Q8IZ83-2 | Isoform 2 of Aldehyde dehydrogenase family 16 member A1 OS=Homo sapiens GN=ALDH16A1 - [A16A1_HUMAN] | 0,77 | no |
| Q9P2E5-2 | Isoform 2 of Chondroitin sulfate glucuronyltransferase OS=Homo sapiens GN=CHPF2 - [CHPF2_HUMAN] | 0,77 | no |
| Q15075 | Early endosome antigen 1 OS=Homo sapiens GN=EEA1 PE=1 SV=2 - [EEA1_HUMAN] | 0,76 | no |
| B4DWR3 | Prefoldin subunit 3 OS=Homo sapiens GN=VBP1 PE=1 SV=1 - [B4DWR3_HUMAN] | 0,76 | no |
| Q96I59-2 | Isoform 2 of Probable asparagine--tRNA ligase, mitochondrial OS=Homo sapiens GN=NARS2 - [SYNM_HUMAN] | 0,76 | no |
| A0A087X033 | Phosphatidylinositol 3,4,5-trisphosphate 3-phosphatase and dual-specificity protein phosphatase PTEN (Fragment) OS=Homo sapiens GN=PTEN PE=1 SV=1 - [A0A087X033_HUMAN] | 0,75 | no |
| Q9NV31 | U3 small nucleolar ribonucleoprotein protein IMP3 OS=Homo sapiens GN=IMP3 PE=1 SV=1 - [IMP3_HUMAN] | 0,74 | no |
| H7C3S9 | COP9 signalosome complex subunit 8 (Fragment) OS=Homo sapiens GN=COPS8 PE=1 SV=1 - [H7C3S9_HUMAN] | 0,73 | no |
| D6RBQ5 | 39S ribosomal protein L3, mitochondrial (Fragment) OS=Homo sapiens GN=MRPL3 PE=1 SV=1 - [D6RBQ5_HUMAN] | 0,72 | no |
| P19623 | Spermidine synthase OS=Homo sapiens GN=SRM PE=1 SV=1 - [SPEE_HUMAN] | 0,72 | no |
| K7ENH2 | Proteasome activator complex subunit 3 (Fragment) OS=Homo sapiens GN=PSME3 PE=1 SV=1 - [K7ENH2_HUMAN] | 0,70 | no |
| P10619-2 | Isoform 2 of Lysosomal protective protein OS=Homo sapiens GN=CTSA - [PPGB_HUMAN] | 0,69 | no |
| Q04446 | 1,4-alpha-glucan-branching enzyme OS=Homo sapiens GN=GBE1 PE=1 SV=3 - [GLGB_HUMAN] | 0,65 | no |
| P49411 | Elongation factor Tu, mitochondrial OS=Homo sapiens GN=TUFM PE=1 SV=2 - [EFTU_HUMAN] | 0,64 | no |
| P37837 | Transaldolase OS=Homo sapiens GN=TALDO1 PE=1 SV=2 - [TALDO_HUMAN] | 0,64 | no |
| B4DJV2 | Citrate synthase OS=Homo sapiens GN=CS PE=1 SV=1 - [B4DJV2_HUMAN] | 0,62 | no |
| Q9BTT0 | Acidic leucine-rich nuclear phosphoprotein 32 family member E OS=Homo sapiens GN=ANP32E PE=1 SV=1 - [AN32E_HUMAN] | 0,62 | no |
| P50135 | Histamine N-methyltransferase OS=Homo sapiens GN=HNMT PE=1 SV=1 - [HNMT_HUMAN] | 0,62 | no |
| A0A087WTH0 | Enolase-phosphatase E1 OS=Homo sapiens GN=ENOPH1 PE=1 SV=2 - [A0A087WTH0_HUMAN] | 0,60 | no |
| Q13151 | Heterogeneous nuclear ribonucleoprotein A0 OS=Homo sapiens GN=HNRNPA0 PE=1 SV=1 - [ROA0_HUMAN] | 0,54 | no |
| Q9H993 | Protein-glutamate O-methyltransferase OS=Homo sapiens GN=ARMT1 PE=1 SV=1 - [ARMT1_HUMAN] | 0,50 | no |
| F5GY32 | tRNA pseudouridine synthase (Fragment) OS=Homo sapiens GN=PUS1 PE=1 SV=2 - [F5GY32_HUMAN] | 0,46 | no |

**Table S3:** **GO functional enrichment analysis of all potential canonical miR-328 targets in microsomal and soluble fraction using STRING v.10.5 database** (medium confidence = 0.4). (A) A GO functional enrichment for all canonical miR-328 targets identified in soluble fraction was found for I.) Biological process and II.) Cellular component. For the enrichment analysis the statistical background “Whole genome” was assumed. For III.) the statistical background “Druggable genome” (6889 genes) was assumed and an enrichment for biological process was recognized. (B) A GO functional enrichment for all canonical miR-328 targets identified microsomal fraction of both proteomics setups was found for I.) Molecular process and II.) Cellular component. The enrichment analysis the statistical background “Whole genome” was assumed. No GO functional enrichment was observed with the statistical background “Druggable genome”.

| **A) Potential canonical miR-328 targets: Soluble fraction** | | | |
| --- | --- | --- | --- |
| **I.) Biological Process (GO)** | | | |
| #pathway ID | pathway description | false discovery rate | matching proteins in your network (labels) |
| GO.0044267 | cellular protein metabolic process | 0.00566 | ALG11,EIF4EBP2,ETF1,IRAK4,MMP2,MRPS35,MTA1,NUP210,PARP16,PCYOX1,PDCD6IP,PHB,PHF15, PPP2R5D,PTPN6,RPS6KA1,RPS6KA3,SLC17A5,SRPRB,SSR1,SSR3,STT3A,SYVN1,TIMM44,TLR2,TMX1, UBE2M,UIMC1,USP4,USP47,YARS |
| GO.0019538 | protein metabolic process | 0.00608 | ALG11,DHCR24,EIF4EBP2,ETF1,HM13,IRAK4,MMP2,MRPS35,MTA1,NUP210,PARP16,PCYOX1,PDCD6IP,  PHB,PHF15,PPP2R5D,TPN6,RPS6KA1,RPS6KA3,SLC17A5,SRPRB,SSR1,SSR3,STT3A,SYVN1,TIMM44,TLR2,  TMX1,UBE2M,UIMC1,USP4,USP47,YARS |
| GO.0044710 | single-organism metabolic process | 0.00608 | ALG11,ATP6V0A2,BCKDHA,CDS2,CERS2,CISD2,CYB5R3,CYBB,ELOVL1,GABARAPL1,GSTM3,HSD17B12,MMP2,MRPS35,MTA1,NT5C,NUP210,PARP16,PCYOX1,PHB,PHF15,PPP2R5D,RPS6KA1,RPS6KA3,SCAP,  SCD,SLC17A5,STT3A,SYVN1,TMX1,TREX1,UIMC1, USP47,YARS |
| GO.0006605 | protein targeting | 0.0114 | ARL6IP1,IPO7,IPO9,SPCS2,SRPRB,SSR1,SSR3,TIMM44,TMED2,TNPO1 |
| GO.0008104 | protein localization | 0.0114 | ARL6IP1,ATP6V0A2,DHCR24,IPO7,IPO9,MON2,MPP7,NUP210,PDCD6IP,SPCS2,SRPRB,SSR1,SSR3,SYVN1,  TIMM44,TLR2,TMED2, TNPO1,USP4 |
| GO.0008152 | metabolic process | 0.0114 | ABCF3,ALG11,ATP2A2,ATP6V0A2,BCKDHA,CDS2,CERS2,CISD2,CYB5R3,CYBB,EIF4EBP2,ELOVL1,ETF1,GABARAPL1,GSTM3,HM13,HSD17B12,IRAK4,MMP2,MRPS35,MTA1,MYO19,NFIA,NOC2L,NT5C,NUP210,  PARP16,PATL1,PCYOX1,PDCD6IP,PHF15,PPP2R5D,PTPN6,RAC2,RPS6KA1,RPS6KA3,SCAP,SCD,SLC17A5,SRPRB,SSR1,SSR3,STT3A,SYVN1,TIMM44,TLR2,TMX1,TNPO1,TREX1,UBE2M,UIMC1,USP4,USP47,YARS |
|  |  |  |  |
| **II.) Cellular Component (GO)** | | | |
| #pathway ID | pathway description | false discovery rate | matching proteins in your network (labels) |
| GO.0005789 | endoplasmic reticulum membrane | 2.82e-14 | AGPAT6,ALG11,ARL6IP1,ATP2A2,CDIPT,CDS2,CERS2,CISD2,CYB5R3,DHCR24,ELOVL1,ESYT1,HM13,  HSD17B12,NUP210,PARP16,REEP6,SCAP, SCD,SRPRB,SSR1,STT3A,SYVN1,TMCO1,TMED2,TMX1,TREX1 |
| GO.0042175 | nuclear outer membrane-endoplasmic reticulum membrane network | 2.82e-14 | AGPAT6,ALG11,ARL6IP1,ATP2A2,CDIPT,CDS2,CERS2,CISD2,CYB5R3,DHCR24,ELOVL1,ESYT1,HM13, HSD17B12,NUP210,PARP16,REEP6,SCAP,SCD, SRPRB,SSR1,STT3A,SYVN1,TMCO1,TMED2,TMX1,TREX1 |
| GO.0044432 | endoplasmic reticulum part | 8.23e-14 | AGPAT6,ALG11,ARL6IP1,ATP2A2,CDIPT,CDS2,CERS2,CISD2,CYB5R3,DHCR24,ELOVL1,ESYT1,HM13, HSD17B12,NUP210,PARP16,PDCD6IP,REEP6,SCAP,SCD,SRPRB,SSR1,STT3A,SYVN1,TMCO1,TMED2, TMX1,TREX1 |
| GO.0005783 | endoplasmic reticulum | 1.52e-12 | AGPAT6,ALG11,ARL6IP1,ATP2A2,CDIPT,CDS2,CERS2,CISD2,CYB5R3,DHCR24,ELOVL1,ESYT1,GABARAPL1,HM13,HSD17B12, MTA1,NUP210,PARP16,PDCD6IP,REEP6,SCAP,SCD,SRPRB,SSR1,STT3A,SYVN1,TMCO1,TMED2,TMEM97,TMX1,TREX1 |
| GO.0031090 | organelle membrane | 1.68e-12 | AGPAT6,ALG11,ARL6IP1,ATAD3B,ATP2A2,ATP6V0A2,CDIPT,CDS2,CERS2,CISD2,CLCN7,CYBB,DHCR24,ELOVL1,ESYT1, GABARAPL1,HM13,HSD17B12,IRAK4,MRPS35,MYO19,NUP210,PARP16,PCYOX1,PHB,RAC2,REEP6,SCAP,SCD,SLC17A5, SLC25A22,SRPRB,SSR1,STT3A,SYVN1,TIMM44,TMCO1,TMED2,TMEM97,TMX1,TREX1 |
|  |  |  |  |
| **III.) Biological Process (GO)** | | | |
| #pathway ID | pathway description | false discovery rate | matching proteins in your network (labels) |
| GO.0034134 | toll-like receptor 2 signaling pathway | 0.0279 | IRAK4,PPP2R5D,RPS6KA1,RPS6KA3,TLR2 |
| GO.0038123 | toll-like receptor TLR1:TLR2 signaling pathway | 0.0279 | IRAK4,PPP2R5D,RPS6KA1,RPS6KA3,TLR2 |
| GO.0038124 | toll-like receptor TLR6:TLR2 signaling pathway | 0.0279 | IRAK4,PPP2R5D,RPS6KA1,RPS6KA3,TLR2 |
| GO.0002755 | MyD88-dependent toll-like receptor signaling pathway | 0.0389 | IRAK4,PPP2R5D,RPS6KA1,RPS6KA3,TLR2 |
| GO.0046341 | CDP-diacylglycerol metabolic process | 0.0389 | AGPAT6,CDIPT,CDS2 |
| GO.0034142 | toll-like receptor 4 signaling pathway | 0.0415 | IRAK4,PPP2R5D,RPS6KA1,RPS6KA3,TLR2 |

| **B) Potential canonical miR-328 targets: Microsomal fraction** | | | |
| --- | --- | --- | --- |
| **I.) Molecular Function (GO)** | | | |
| #pathway ID | pathway description | false discovery rate | matching proteins in your network (labels) |
| GO.0005524 | ATP binding | 0.0296 | AARS2,ABCF3,ABL2,ADCK3,ATAD3B,ATP2A2,CDC42BPB,CDK8,DHX37,EHD2,IP6K1,IRAK4,KIF13B, MAP3K11,RFC5,RPS6KA1,RPS6KA3,TRAP1 |
| GO.0035639 | purine ribonucleoside triphosphate binding | 0.0296 | AARS2,ABCF3,ABL2,ADCK3,ATAD3B,ATP2A2,CDC42BPB,CDK8,DHX37,EHD2,IP6K1,IRAK4,IRGQ, KIF13B,MAP3K11,RFC5,RHOB,RPS6KA1,RPS6KA3,TRAP1 |
| GO.0003824 | catalytic activity | 0.041 | AARS2,ABCF3,ABL2,ADCK3,AGPAT5,ATG4B,ATP2A2,CDC42BPB,CPT1A,DHX37,EHD2,ELOVL1,ERI3, GSTM3,HM13,HMOX2,IP6K1,IRAK4,ISOC2,KIAA0448,KIF13B,MIB1,PLCB1,POLR2E,PRMT1,PTPRF,PTPRJ,RHOB,RNF126,RPS6KA1,RPS6KA3,SCD,STT3A,SUMO3,VCPIP1,ZADH2 |
| GO.0032550 | purine ribonucleoside binding | 0.041 | AARS2,ABCF3,ABL2,ADCK3,ATAD3B,ATP2A2,CDC42BPB,CDK8,DHX37,EHD2,IP6K1,IRAK4,IRGQ, KIF13B,MAP3K11,RFC5,RPS6KA1,RPS6KA3,TRAP1 |
| GO.0032555 | purine ribonucleotide binding | 0.041 | AARS2,ABCF3,ABL2,ADCK3,ATAD3B,ATP2A2,CDC42BPB,CDK8,DHX37,EHD2,IP6K1,IRAK4,IRGQ, KIF13B,MAP3K11,RFC5,RPS6KA1,RPS6KA3,TRAP1 |
| GO.0043168 | anion binding | 0.041 | AARS2,ABCF3,ABL2,ADCK3,ATAD3B,ATP2A2,CD300A,CDC42BPB,CDK8,DHX37,EHD2,GSTM3,IP6K1, IRAK4,IRGQ,KIF13B,MAP3K11,PLCB1,PTPRF,RFC5,RPS6KA1,RPS6KA3,TRAP1,ZFYVE26 |
|  |  |  |  |
| **II.) Cellular Component (GO)** | | | |
| #pathway ID | pathway description | false discovery rate | matching proteins in your network (labels) |
| GO.0044422 | organelle part | 2.94e-06 | AGPAT5,ATAD3B,ATP2A2,BCL2L1,BMI1,CDC42BPB,CDK8,DHX37,EHD2,ELOVL1,ERI3,FERMT3,GPSM1, GSTM3,HAUS4,HAUS5,HM13,HMOX2,IP6K1,IRAK4,IVNS1ABP,JAGN1,KIAA0448,KIF13B,MAP3K11, MED22,MIB1,MLST8,MON2,MRPL10,MTCH1,PLCB1,POLR2E,PRMT1,PSAP,PSMD1,PTPRJ,PUF60, RAP1GAP2,RFC5,RHOB,RPS6KA3,SCD,SLC25A22,SLC25A3,STARD3,STT3A,SUMO3,TMED2,TMEM97, TOMM40L,TRAP1,UBP1,UCHL5,VCPIP1,ZCCHC17,ZFYVE26 |
| GO.0044446 | intracellular organelle part | 2.94e-06 | AGPAT5,ATAD3B,ATP2A2,BCL2L1,BMI1,CDC42BPB,CDK8,DHX37,EHD2,ELOVL1,ERI3,FERMT3,GPSM1, HAUS4,HAUS5,HM13,HMOX2,IP6K1,IRAK4,IVNS1ABP,JAGN1,KIAA0448,KIF13B,MAP3K11,MED22,MIB1,MLST8,MON2,MRPL10,MTCH1,PLCB1,POLR2E,PRMT1,PSAP,PSMD1,PTPRJ,PUF60,RAP1GAP2,RFC5, RHOB,RPS6KA3,SCD,SLC25A22,SLC25A3,STARD3,STT3A,SUMO3,TMED2,TMEM97,TOMM40L,TRAP1, UBP1,UCHL5,VCPIP1,ZCCHC17,ZFYVE26 |
| GO.0005737 | cytoplasm | 3.41e-05 | AARS2,ABL2,ADCK3,AGPAT5,ARHGAP1,ATAD3B,ATG4B,ATP2A2,BCL2L1,BMI1,CDC42BPB,DHX37, ELOVL1,ERI3,GPSM1,GSTM3,HAUS4,HAUS5,HM13,HMOX2,IP6K1,IPO9,IRAK4,ISOC2,IVNS1ABP,JAGN1, KIAA0448,KIF13B,MAP3K11,MED22,MLST8,MON2,MRPL10,MTCH1,PLCB1,POLR2E,PRMT1,PSMD1, PSTPIP2,PUF60,RAP1GAP2,RASGRP2,RHOB,RNF126,RPS6KA1,RPS6KA3,SCD,SLC25A22,SLC25A3,STT3A,SUMO3,TBCEL,TMED2,TMEM65,TMEM97,TNPO1,TNPO2,TOMM40L,TRAP1,UBP1,UCHL5,VCPIP1, ZFYVE26 |
| GO.0031090 | organelle membrane | 0.00211 | AGPAT5,ATAD3B,ATP2A2,BCL2L1,EHD2,ELOVL1,GPSM1,HM13,HMOX2,IRAK4,JAGN1,KIAA0448, MRPL10,MTCH1,PLCB1,PSAP,RAP1GAP2,RHOB,SCD,SLC25A22,SLC25A3,STARD3,STT3A,TMED2, TMEM97,TOMM40L,TRAP1,ZFYVE26 |
| GO.0043229 | intracellular organelle | 0.0022 | AARS2,ABL2,ADCK3,AGPAT5,ATAD3B,ATP2A2,BCL2L1,BMI1,CDC42BPB,CDK8,DHX37,EHD2,ELOVL1, ERI3,FERMT3,GPSM1,GSTM3,HAUS4,HAUS5,HM13,HMOX2,IP6K1,IPO9,IRAK4,ISOC2,JAGN1,KIAA0448, KIF13B,MAP3K11,MED22,MLST8,MON2,MRPL10,MTCH1,PLCB1,POLR2E,PRMT1,PSMD1,PSTPIP2,PTPRJ, PUF60,RAP1GAP2,RFC5,RNF126,RPS6KA3,SCD,SLC25A22,SLC25A3,STT3A,SUMO3,TBCEL,TMED2, TMEM65,TMEM97,TNPO1,TNPO2,TOMM40L,TRAP1,UBP1,UCHL5,VCPIP1,ZCCHC17,ZFYVE26 |

**Table S4:** **GO functional enrichment analysis of all potential non-canonical miR-328 targets in microsomal and soluble fraction using STRING v.10.5 database** (medium confidence = 0.4). (A) A GO functional enrichment for all non-canonical miR-328 targets identified in soluble fraction was found for I.) Biological process and II.) Cellular component and III.) Molecular function. For the enrichment analysis the statistical background “Whole genome” was assumed. No GO functional enrichment was observed with the statistical background “Druggable genome” (6889 genes).(B) A GO functional enrichment for all non-canonical miR-328 targets identified microsomal fraction of both proteomics setups was found for I.) Biological Process, II.) Cellular component and III.) Molecular function. The enrichment analysis the statistical background “Whole genome” was assumed.

| **A) Potential non-canonical miR-328 targets: Soluble fraction** | | | |
| --- | --- | --- | --- |
| **I.) Biological Process (GO)** | | | |
| #pathway ID | pathway description | false discovery rate | matching proteins in your network (labels) |
| GO.0006397 | mRNA processing | 0.0421 | AKAP17A,DCPS,HNRNPA0,HNRNPH2,PHRF1,PPIL1,PRPF40A,SF3A2,SNRNP70 |
| GO.0044237 | cellular metabolic process | 0.0421 | AACS,AKAP17A,APEX1,ASS1,BCAS3,COX6B1,CTSA,DOHH,DYNLL1,EEF1D,ENOPH1,FDPS,GPCPD1, GSTM3,HMGN5,HNRNPA0,HNRNPH2,IRF2BP1,LPPR3,MTHFD2,NDUFA2,NEK3,NSMAF,NUDT2,NUDT3, PBX2,PELO,PHRF1,PPIL1,PPM1A,PRPF40A,PSPH,RNF10,SENP1,SETD7,SF3A2,SNRNP70,TEC,TRNT1, TUBB4B,TUFM,UBA1,UBE2F,UBLCP1 |
| GO.0008380 | RNA splicing | 0.0459 | AKAP17A,DCPS,HNRNPA0,HNRNPH2,PPIL1,PRPF40A,SF3A2,SNRNP70 |
| GO.0034641 | cellular nitrogen compound metabolic process | 0.0459 | AKAP17A,ANXA1,APEX1,ASS1,BCAS3,COX6B1,DYNLL1,EEF1D,ENOPH1,GSTM3,HMGN5,HNRNPA0, HNRNPH2,IRF2BP1, NDUFA2,NSMAF,NUDT2,NUDT3,PBX2,PELO,PHRF1,PPIL1,PPM1A,PRPF40A,PSPH,RFK,RNF10,SETD7, SF3A2,SNRNP70, TRNT1,TUFM |
| GO.0044238 | primary metabolic process | 0.0459 | AACS,AKAP17A,APEX1,ASS1,BCAS3,COX6B1,DOHH,DYNLL1,EEF1D,ENOPH1,FASTKD5,FDPS,GPCPD1, HMGN5,HNRNPA0,HNRNPH2,IRF2BP1,NDUFA2,NEK3,NLRP1,NSMAF,NUDT2,NUDT3,PBX2,PELO,PHRF1,PPIL1,PPM1A,PRPF40A,PSPH,RFK, RNF10,SENP1,SETD7,SF3A2,SNRNP70,TEC,TRNT1,TUBB4B,TUFM,UBA1,UBE2F,UBLCP1 |
| GO.0071704 | organic substance metabolic process | 0.0459 | AACS,AKAP17A,APEX1,ASS1,BCAS3,COX6B1,DOHH,DYNLL1,EEF1D,ENOPH1,FASTKD5,FDPS,GPCPD1, GSTM3,HMGN5,HNRNPA0,HNRNPH2,IRF2BP1,NDUFA2,NEK3,NLRP1,NSMAF,NUDT2,NUDT3,PBX2, PELO,PHRF1,PPIL1,PPM1A,PRPF40A,PSPH,RFK,RNF10,SENP1,SETD7,SF3A2,SNRNP70,TEC,TRNT1, TUBB4B,TUFM,UBA1,UBE2F,UBLCP1 |
|  |  |  |  |
| **II.) Cellular Component (GO)** | | | |
| #pathway ID | pathway description | false discovery rate | matching proteins in your network (labels) |
| GO.0044424 | intracellular part | 0.000564 | AACS,AKAP17A,ANXA1,ANXA4,APEX1,BCAS3,C21orf59,CCDC50,COX6B1,CS,CTSA,DCPS,DOHH, DYNLL1,EEF1D,ENOPH1,FASTKD5,FDPS,GNL3L,GPCPD1,GSTM3,HNRNPA0,HNRNPH2,IRF2BP1, MAP3K11,MTHFD2,NDUFA2,NEK3,NLRP1,NSMAF,NUDT2,NUDT3,PELO,PLCG2,POLR1B,POTEI,PPIL1, PPM1A,PRCP,PRPF40A,PSPH,RBM12,RCC1,RNASEH2B,RNF10,SENP1,SETD7,SF3A2,SNRNP70,SYK,TEC, TRNT1,TUBB4B,TUFM,UBA1,UBE2F,UBE2G1,UBLCP1,WIZ |
| GO.0005622 | intracellular | 0.00093 | AACS,AKAP17A,ANXA1,ANXA4,APEX1,BCAS3,C21orf59,CCDC50,COX6B1,CS,CTSA,DCPS,DOHH,DYNLL1,EEF1D,ENOPH1,FASTKD5,FDPS,GNL3L,GPCPD1,GSTM3,HNRNPA0,HNRNPH2,IRF2BP1,MAP3K11, MTHFD2,NDUFA2,NEK3,NLRP1,NSMAF,NUDT2,NUDT3,PELO,PLCG2,POLR1B,POTEI,PPIL1,PPM1A,PRCP, PRPF40A,PSPH,RBM12,RCC1,RNASEH2B,RNF10,SENP1,SETD7,SF3A2,SNRNP70,SYK,TEC,TRNT1, TUBB4B,TUFM,UBA1,UBE2F,UBE2G1,UBLCP1,WIZ |
| GO.0005634 | nucleus | 0.002 | AKAP17A,ANXA1,ANXA4,APEX1,ASS1,BCAS3,C21orf59,CS,CTSA,DCPS,DYNLL1,EEF1D,ENOPH1,FDPS, GNL3L,GSTM3,HNRNPA0,HNRNPH2,IRF2BP1,NEK3,NLRP1,PBX2,PELO,POLR1B,PPIL1,PPM1A,PRPF40A, RBM12,RCC1,RNASEH2B,RNF10,SENP1,SETD7,SF3A2,SNRNP70,SYK,TUBB4B,UBA1,UBLCP1,WIZ |
| GO.0043229 | intracellular organelle | 0.00292 | AKAP17A,ANXA1,ANXA4,APEX1,ASS1,BCAS3,C21orf59,COX6B1,CS,CTSA,DCPS,DYNLL1,EEF1D,ENOPH1,FASTKD5,FDPS,GNL3L,GSTM3,HNRNPA0,HNRNPH2,IRF2BP1,MAP3K11,MTHFD2,NDUFA2,NEK3, NLRP1,NUDT2,PBX2,PELO,POLR1B,POTEI,PPIL1,PPM1A,PRCP,PRPF40A,RAP2C,RBM12,RCC1,RFK, RNASEH2B,RNF10,SENP1,SETD7,SF3A2,SNRNP70,SYK, TEC, TRNT1,TUBB4B,TUFM,UBA1,UBLCP1,WIZ |
| GO.0043231 | intracellular membrane-bounded organelle | 0.00537 | AKAP17A,ANXA1,ANXA4,APEX1,ASS1,BCAS3,C21orf59,COX6B1,CS,CTSA,DCPS,DYNLL1,EEF1D, ENOPH1,FASTKD5,FDPS,GNL3L,GSTM3,HNRNPA0,HNRNPH2,IRF2BP1,MTHFD2,NDUFA2,NEK3,NLRP1, NUDT2,PBX2,PELO,POLR1B,PPIL1,PPM1A,PRCP,PRPF40A,RAP2C,RBM12,RCC1,RFK,RNASEH2B,RNF10, SENP1,SETD7,SF3A2,SNRNP70,SYK,TRNT1,TUBB4B,TUFM, UBA1,UBLCP1,WIZ |
|  |  |  |  |
| **III.) Molecular Function (GO)** | | | |
| #pathway ID | pathway description | false discovery rate | matching proteins in your network (labels) |
| GO.0003824 | catalytic activity | 1.68e-06 | AACS,ANXA1,APEX1,ASS1,COX6B1,CS,DCPS,DOHH,DYNLL1,ENOPH1,FASTKD5,FDPS,GNL3L,GPCPD1, GSTM3,IRF2BP1,LPPR3,MTHFD2,NDUFA2,NEK3,NUDT2,NUDT3,PELO,PLCG2,POLR1B,PPIL1,PPM1A, PRCP,PSPH,RFK,RNASEH2B,RNF10,SENP1,SETD7,TEC,TRNT1,TUBB4B,TUFM,UBA1,UBE2F,UBLCP1 |
| GO.0003723 | RNA binding | 2.19e-05 | AKAP17A,ANXA1,APEX1,ASS1,CS,DCPS,EEF1D,FASTKD5,FDPS,GNL3L,HMGN5,HNRNPA0,HNRNPH2, PRPF40A,RBM12, SF3A2,SNRNP70,TRNT1,TUBB4B,TUFM,UBA1 |
| GO.0000166 | nucleotide binding | 0.000701 | AACS,AKAP17A,ASS1,GNL3L,HNRNPA0,HNRNPH2,MAP3K11,NEK3,NLRP1,NUDT2,POTEI,RAP2C,RBM12,RFK,SNRNP70, SYK,TEC,TRNT1,TUBB4B,TUFM,UBA1,UBE2F,UBE2G1 |
| GO.0044822 | poly(A) RNA binding | 0.00071 | AKAP17A,APEX1,ASS1,CS,FASTKD5,FDPS,GNL3L,HMGN5,HNRNPA0,HNRNPH2,PRPF40A,RBM12,SF3A2,SNRNP70,TUFM,UBA1 |
| GO.0036094 | small molecule binding | 0.000796 | AACS,AKAP17A,ASS1,GNL3L,HNRNPA0,HNRNPH2,MAP3K11,NEK3,NLRP1,NUDT2,POLR1B,POTEI,RAP2C,RBM12,RFK, SNRNP70,SYK,TEC,TRNT1,TUBB4B,TUFM,UBA1,UBE2F,UBE2G1 |

| **B) Potential non-canonical miR-328 targets: Microsomal fraction** | | | |
| --- | --- | --- | --- |
| **I.) Biological process (GO)** | | | |
| #pathway ID | pathway description | false discovery rate | matching proteins in your network (labels) |
| GO.0045806 | negative regulation of endocytosis | 0.0363 | APOC3,HMGB1,LRSAM1,PACSIN2,TGFB1 |
|  |  |  |  |
| **II.) Cellular Compartment (GO)** | | | |
| #pathway ID | pathway description | false discovery rate | matching proteins in your network (labels) |
| GO.0005634 | nucleus | 0.000532 | ANAPC13,ANKRD17,ANP32E,ANXA1,ANXA4,ARHGDIA,ARNT,ASRGL1,BAG4,BANF1,BRD1,CDK12,CTSA,CTSB,DDX28, DIS3L2,DYNLL2,EBNA1BP2,ENSA,FAM103A1,FDPS,FHIT,FTH1,GSTM3,HMGB1,HNMT,HNRNPA0,IFT74,IMP3,LRIF1,MRPL1,NOP16,NPEPL1,PACSIN2,PAK1IP1,POLR2I,PPM1A,RBM6,RNF10,RPS19,S100A4,S100A9,SGOL2,STRBP,SYK,TACC1, TALDO1,TGFB1,TIMELESS,TNKS1BP1,TRIM21,TROVE2,TTI2,UBA1,UBAP2L,UIMC1,USP36,WDR55 |
| GO.0043227 | membrane-bounded organelle | 0.000532 | ACY1,AKR1A1,ALDH16A1,ANAPC13,ANKRD17,ANP32E,ANXA1,ANXA4,APOC3,ARNT,ASRGL1,BAG4,BANF1,BDH2,BRD1, C14orf2,CAP1,CDK12,CHPF2,CMC2,COX6B1,CTSA,DDX28,DIS3L2,DSC2,DYNLL2,EBNA1BP2,EEA1,ENSA,FAM103A1,FDPS,FHIT,FTH1,GBE1,GC,GCSH,GSTM3,HMGB1,HNMT,HNRNPA0,HSPE1,IMP3,KTN1,LRIF1,MPV17,MRPL1,NARS2,NDUFA2, NOP16,NPEPL1,OGDH,PACSIN2,PAK1IP1,POLR2I,PPM1A,PRCP,PTER,RBM6,RNF10,RPS19,S100A4,S100A9,SGOL2,STRBP,SYK,TACC1,TALDO1,TGFB1,TIMELESS,TNKS1BP1,TRIM21,TROVE2,TTI2,TUFM,UBA1,UBAC2,UBAP2L,UIMC1,USP36,VTN,WDR55 |
| GO.0070062 | extracellular exosome | 0.000532 | ACY1,AKR1A1,ALDH16A1,ANXA1,ANXA4,APOC3,ARHGDIA,BANF1,BDH2,CAP1,CTSA,CTSB,DBI,DSC2,EEA1,FHIT,FTH1, GBE1,GC,GSTM3,HNMT,HSPE1,PACSIN2,PRCP,PTER,PYGM,RPS19,S100A4,S100A9,TALDO1,TUFM,UBA1,VTN |
| GO.0031988 | membrane-bounded vesicle | 0.000751 | ACY1,AKR1A1,ALDH16A1,ANP32E,ANXA1,ANXA4,APOC3,ARHGDIA,BANF1,BDH2,CAP1,CTSA,CTSB,DBI,DSC2,EEA1,FHIT, FTH1,GBE1,GC,GSTM3,HNMT,HSPE1,IFT74,PACSIN2,PRCP,PTER,PYGM,RPS19,S100A4,S100A9,SYK,TALDO1,TGFB1, TUFM,UBA1,VTN |
| GO.0044424 | intracellular part | 0.00104 | ACY1,AKR1A1,ANAPC13,ANKRD17,ANP32E,ANXA1,ANXA4,APOC3,ARNT,ASRGL1,BAG4,BANF1,BDH2,BRD1,C14orf2,CAP1,CDK12,CEP55,CHPF2,CMC2,COX6B1,CRIP1,CTSA,DDX28,DIS3L2,DSC2,DYNLL2,EBNA1BP2,ENSA,FAM103A1,FDPS,FHIT , FTH1,GBE1,GCSH,GPCPD1,GSTM3,HMGB1,HNMT,HNRNPA0,HSPE1,IMP3,KTN1,LATS1,LRIF1,LRSAM1,MPV17,MRPL1, NARS2,NDUFA2,NOP16,OGDH,PAK1IP1,PHACTR4,POLR2I,PPM1A,PRCP,RBM6,RCSD1,RNF10,RPS19,S100A4,S100A9, SGOL2,SHFM1,SRM,SYK,TACC1,TALDO1,TGFB1,TIMELESS,TNKS1BP1,TRIM21,TROVE2,TTI2,TUFM,UBA1,UBAC2,UBAP2L,UIMC1,UPP1,USP36,WDR55,ZFAND6 |
| **III.) Molecular Function (GO)** | | | |
| #pathway ID | pathway description | false discovery rate | matching proteins in your network (labels) |
| GO.0044822 | poly(A) RNA binding | 9.19e-05 | ANKRD17,BAG4,DDX28,EBNA1BP2,FAM103A1,FDPS,HMGB1,HNRNPA0,HSPE1,IMP3,KTN1,MRPL1,NOP16,RBM6,RPS19,S100A4,STRBP,TUFM,UBA1,UBAP2,UBAP2L,USP36,YTHDC1 |
| GO.0003723 | RNA binding | 0.000409 | ANKRD17,ANXA1,BAG4,DDX28,DIS3L2,EBNA1BP2,FAM103A1,FDPS,HMGB1,HNRNPA0,HSPE1,IMP3,KTN1,MRPL1,NOP16,RBM6,RPS19,S100A4,TRIM21,TUFM,UBA1,UBAP2,UBAP2L,USP36,YTHDC1 |
| GO.0050786 | RAGE receptor binding | 0.017 | HMGB1,S100A4,S100A9 |
|  |  |  |  |
| **IV.) Biological Process (GO)** | | | |
| #pathway ID | pathway description | false discovery rate | matching proteins in your network (labels) |
| GO.0031988 | membrane-bounded vesicle | 0.0214 | ACY1,AKR1A1,ALDH16A1,ANP32E,ANXA1,ANXA4,APOC3,ARHGDIA,BANF1,BDH2,CAP1,CTSA,CTSB,DBI,DSC2,EEA1, FHIT,FTH1,GBE1,GC,GSTM3,HNMT,HSPE1,IFT74,PACSIN2,PRCP,PTER,PYGM,RPS19,S100A4,S100A9,SYK,TALDO1, TGFB1,TUFM,UBA1,VTN |
| GO.0070062 | extracellular exosome | 0.0214 | ACY1,AKR1A1,ALDH16A1,ANXA1,ANXA4,APOC3,ARHGDIA,BANF1,BDH2,CAP1,CTSA,CTSB,DBI,DSC2,EEA1,FHIT,FTH1, GBE1,GC,GSTM3,HNMT,HSPE1,PACSIN2,PRCP,PTER,PYGM,RPS19,S100A4,S100A9,TALDO1,TUFM,UBA1,VTN |
